# A new generation of nanobody research tools using improved mass spectrometry-based discovery methods

**DOI:** 10.1016/j.jbc.2024.107623

**Published:** 2024-08-02

**Authors:** Peter C. Fridy, Ryan J. Farrell, Kelly R. Molloy, Sarah Keegan, Junjie Wang, Erica Y. Jacobs, Yinyin Li, Jill Trivedi, Viren Sehgal, David Fenyö, Zhuhao Wu, Brian T. Chait, Michael P. Rout

**Affiliations:** 1 Laboratory of Cellular and Structural Biology, The Rockefeller University, New York, New York, USA; 2 Laboratory of Brain Development and Repair, The Rockefeller University, New York, New York, USA; 3 Department of Biochemistry, Weill Cornell Medicine, New York, New York, USA; 4 Laboratory of Mass Spectrometry and Gaseous Ion Chemistry, The Rockefeller University, New York, New York, USA; 5 Department of Biochemistry and Molecular Pharmacology, Institute for Systems Genetics, NYU Grossman School of Medicine, New York, New York, USA; 6 Chemistry Department, St John's University, Queens, New York, USA

**Keywords:** single-domain antibody, monoclonal antibody, antibody engineering, mass spectrometry, immunohistochemistry

## Abstract

Single-domain antibodies (“nanobodies”) derived from the variable region of camelid heavy-chain only antibody variants have proven to be widely useful tools for research, therapeutic, and diagnostic applications. In addition to traditional display techniques, methods to generate nanobodies using direct detection by mass spectrometry and DNA sequencing have been highly effective. However, certain technical challenges have limited widespread application. We have optimized a new pipeline for this approach that greatly improves screening sensitivity, depth of antibody coverage, antigen compatibility, and overall hit rate and affinity. We have applied this improved methodology to generate significantly higher affinity nanobody repertoires against widely used targets in biological research—*i.e.*, GFP, tdTomato, GST, and mouse, rabbit, and goat immunoglobulin G. We have characterized these reagents in affinity isolations and tissue immunofluorescence microscopy, identifying those that are optimal for these particularly demanding applications, and engineering dimeric constructs for ultra-high affinity. This study thus provides new nanobody tools directly applicable to a wide variety of research problems, and improved techniques enabling future nanobody development against diverse targets.

Nanobodies are single-domain, antigen-specific proteins derived from the variable domain (V_H_H) of a heavy chain only immunoglobulin G (IgG) antibody variant (HCAb) found in camelid species such as llamas, alpacas, and camels (1). Though approximately one-tenth the size of typical antibodies, nanobodies retain comparable antigen affinity and specificity, can access novel antigenic sites, and can be readily cloned and expressed recombinantly in bacteria (2, 3, 4). In comparison with monoclonal antibodies, they possess numerous important advantages, including increased stability, tissue penetrance, solubility, and high amenability to genetic or chemical engineering (5, 6). These advantages have made nanobodies an increasingly popular option for therapeutic, diagnostic, and research applications that demand targeted high-affinity reagents (7, 8).

Nanobodies can typically be generated from immunized camelids through phage display technology (9, 10), yeast display (11, 12, 13), or through direct identification by mass spectrometry (MS) combined with high-throughput DNA sequencing (14, 15, 16). The MS-based pipeline for nanobody identification has been successfully used to generate large repertoires of high-quality reagents, and has significant advantages in stringency, speed, and lack of an intermediate library; however, certain technical demands have limited the applications and widespread adoption of this approach. We therefore sought to significantly improve the ease of this technology as well as the quality of its resulting nanobody candidates, allowing it to be applied with high success rates to a much wider range of target antigens. We made improvements throughout our pipeline including in the methods used for serum antibody sample preparation, in the MS analysis of the resulting purified V_H_H mixtures, and in the bioinformatics software used for candidate identification and ranking. We also significantly improved the coverage of V_H_H sequences from lymphocyte complementary DNA (cDNA) by optimizing the primers used for PCR amplification; this optimization is widely applicable to nanobody identification approaches, as V_H_H amplification is also a key step in preparing display libraries from either naive or immunized animals.

As examples from our improved MS-based method, we report new or improved nanobodies against several antigens of broad research use, including the fluorescent protein tags GFP and tdTomato, the expression fusion protein GST tag, as well as IgG from mouse, rabbit, and goat. These nanobodies are ultra-high affinity, with K_D_s into the sub-nanomolar range, and are suitable for high-quality immunoprecipitation and immunofluorescence (IF) applications.

## Results

**Figure 1:**
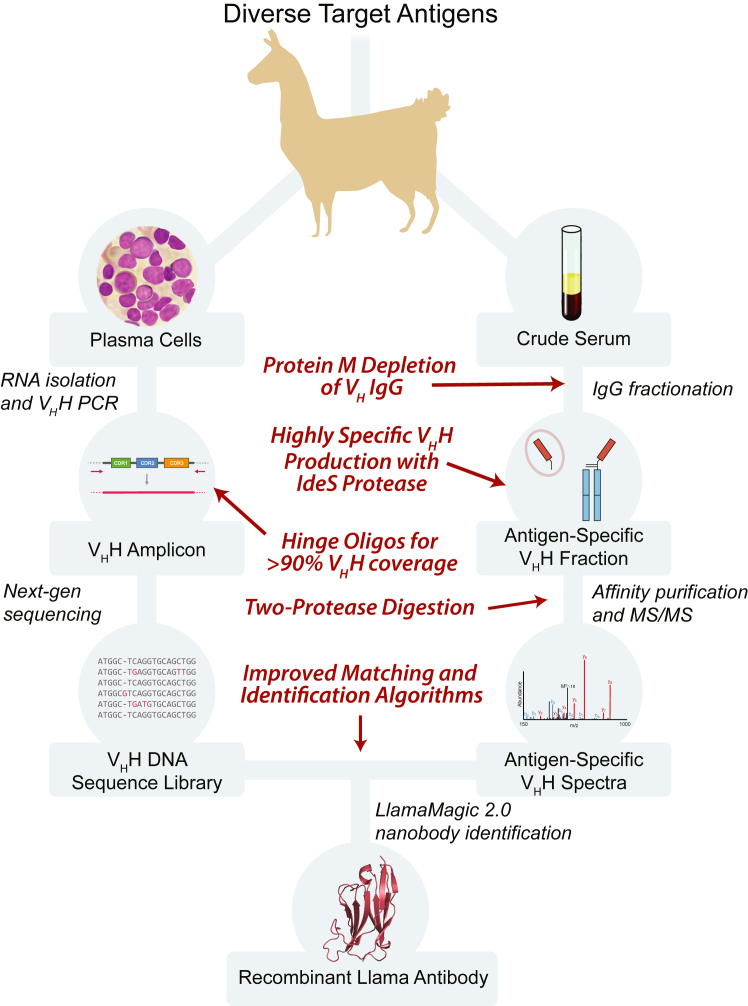
**Improved pipeline for nanobody generation by MS and next-gen DNA sequencing.** Overview of the methodology used to identify nanobody candidates from immunized llamas, with major improvements indicated in *red*. MS, mass spectrometry.

Our new methods for nanobody identification build off our previously published pipeline and rely on similar parallel procedures (Fig. 1). First, the variant HCAb of interest is purified from crude serum taken from an immunized camelid. The target antigen is then immobilized and used to affinity-isolate antigen-specific HCAb from this fraction. Bound HCAb is protease-treated on the antigen resin to release the Fc domains, and the remaining bound V_H_H is eluted and resolved by SDS-PAGE. V_H_H-specific proteins are digested and analyzed by liquid chromatography tandem MS to generate peptide sequence data. In parallel to this serum analysis, RNA is extracted from lymphocytes purified from the bone marrow sampled from the same animal. Expressed V_H_H sequences from this RNA are then amplified by RT-PCR to produce a cDNA library for Illumina sequencing. Antigen-specific nanobodies are identified by using our Llama-Magic software (https://github.com/FenyoLab/Llama_Nanobodies) to search the library with the peptide sequence data. All steps of these parallel procedures were optimized from their original versions for improved nanobody identification and robustness to sample variability.

### Improvements in the identification of nanobodies from llama sera

**Figure 2:**
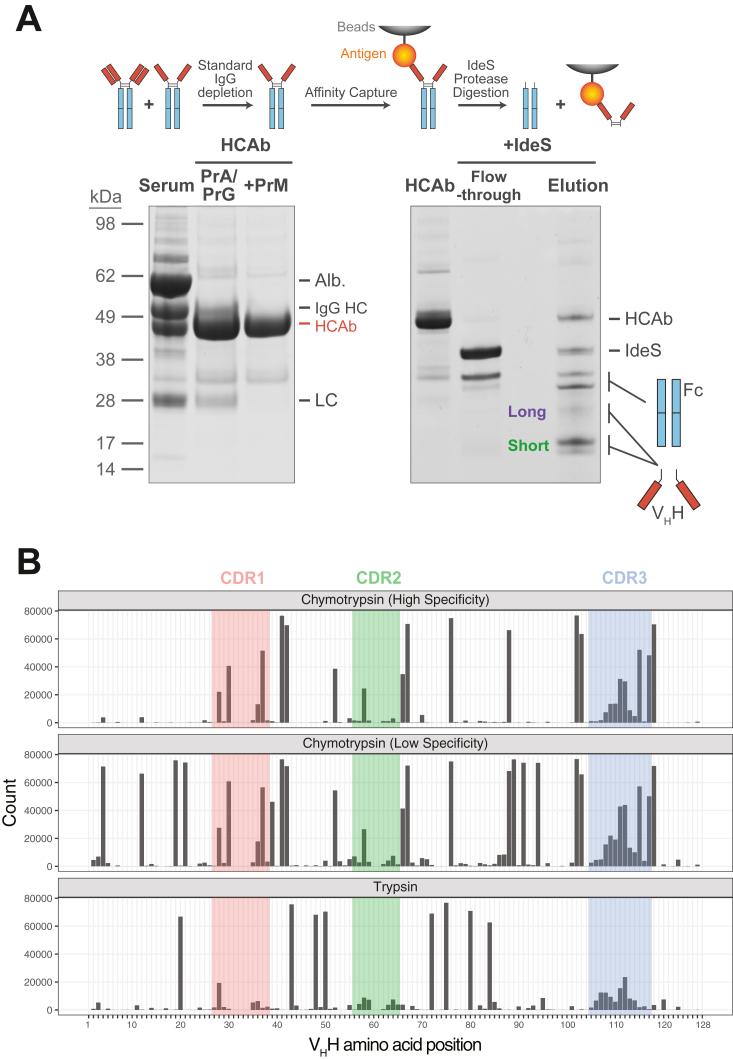
**Improvements in V**_**H**_**H isolation and digestion for MS.***A*, representative purification of HCAb using Protein A, G, and M (*left*), and IdeS digestion after affinity isolation (*right*). Coomassie-stained SDS-PAGE gels are shown, with bands labeled based on dominant protein as identified by MS. Crude serum input (“Serum”) was purified over Protein A and Protein G for initial HCAb enrichment (“PrA/PrG”) at 94 ± 0.8% purity (n = 5). A second step depletion over PrM (“+PrM”) results in a high purity HCAb fraction: HCAb yield was 89 ± 2.2%, with standard IgG not measurable above background (n = 5). After affinity capture of HCAb on antigen resin (*right*), IdeS was added to digest bound HCAb. After digestion, cleaved Fc and IdeS are released in the reaction solution (“Flow-through”). Remaining antigen-bound fragments are then eluted by boiling in SDS (“Elution”). *B*, *in silico* protease digestion was performed against an experimental llama V_H_H sequence library. Counts of protease cleavage events at a given residue are plotted, using IMGT IgG amino acid numbering, with CDRs highlighted in *color*. For chymotrypsin, “low-specificity” predictions include potential cleavages after Met, Leu, and His residues, in addition to “high-specificity” cleavages after Phe, Tyr, and Trp. CDR, complementarity determining region; IgG, immunoglobulin G.

Direct identification of nanobody candidates from camelid serum relies on MS analysis of highly purified antigen-specific HCAb protein. Previously, HCAb fractions have been obtained from crude serum by selective binding to and elution from Protein A and Protein G resin. While this results in significant enrichment of HCAb, IgG contamination is typical in this preparation (5–15%) (14), interfering with MS analysis by introducing irrelevant peptides and complexity. We took advantage of an additional IgG-binding factor from *mycoplasma*, protein M, which specifically targets light chain subunits and should therefore bind standard IgG but not HCAb (17). As predicted, passing our samples over protein M resin led to virtually complete depletion of IgG (Fig. 2*A*). By incorporating this depletion step into sample preparation upstream of MS, we essentially eliminate this source of contamination.

Another key concern in the quality of protein purified for MS is the removal of Fc, which otherwise increases spectral complexity and complicates V_H_H peptide identification. This was initially done by a traditional papain digest of HCAb captured on antigen resin. While this effectively removes Fc, papain is a relatively nonspecific protease, and depending on the antigen used for purification it can be difficult or impossible to tune digestion conditions to cleave off Fc without excessive degradation of either the immobilized antigen or the V_H_H fragment itself. As an alternative, we surveyed sequence-specific proteases and identified the streptococcal protease IdeS as a superior option for Fc/F(ab’)_2_ digestion of llama or alpaca HCAb (18). Compared to papain across a variety of protein standards, IdeS digestion readily cleaves IgG with no off-target proteolysis (Fig. S1*A*). Moreover, when used on affinity-purified llama HCAb, IdeS treatment produces distinct bands correlating to Fc and V_H_H domains which are readily separated by SDS-PAGE for MS analysis (Fig. 2*A*). In comparing MS samples prepared from serum previously used to identify anti-GFP nanobody candidates (14), both IdeS and papain performed equally better than no Fc digestion, but IdeS without concomitant off-target digestion issues (Fig. S1, *A* and *B*).

### Optimization of V_H_H mass spectrometric analysis

Nanobody sequences are identified by computationally matching peptides to full-length protein sequences identified from *in silico* translation of cDNA, and so adequate peptide coverage is vital to high-confidence candidate selection. Peptides covering the complementarity determining region 3 (CDR3) are particularly important, as this is the most divergent and defining region of each species of V_H_H (19). Multiple improvements were thus made to our MS analyses to improve peptide identification and sequence matching, as follows. First, SDS-PAGE-resolved and Coomassie blue-stained V_H_H bands were excised from the separating gel, split in half, and each digested in parallel with either trypsin or chymotrypsin, rather than with trypsin alone. As previously observed, regions adjacent to CDR3 were more favored for cleavage by chymotrypsin than trypsin (Fig. 2*B*) (15). Trypsin nevertheless provided superior V_H_H coverage overall, and we found that integrating MS data from parallel trypsin and chymotrypsin samples provided the most comprehensive sequence coverage (Fig. S1*C*).

Additionally, we introduced an optional subslicing of SDS-PAGE separated V_H_H bands before MS to enable greater correlation between peptides derived from a single V_H_H protein. A major challenge of MS identification of sequences from a complex antibody mixture is the need to identify and correlate the three associated CDRs, separated by highly conserved framework regions, to define a unique antibody sequence; thus, for high confidence identification, peptides covering the different CDRs must be correctly matched to a single origin protein. To improve this correlation, the V_H_H gel region can be sliced horizontally to generate several sections for digestion and MS analysis, significantly decreasing the complexity of each sample and limiting incorrect peptide matches across different V_H_H proteins (Fig. S1*D*).

In cases where affinity isolations have yielded several micrograms of V_H_H protein, we have also used offline high pH reversed phase peptide fractionation to decrease the complexity of each sample subjected to mass spectrometry. With a decrease in the chromatographic density of each fraction and an increase in the overall amount of sample that can be analyzed by LC-MS, fractionation enhances the sensitivity of peptide identification and therefore improved V_H_H sequence coverage, as previously established in other MS workflows (20).

### Using RNA-seq to redesign PCR amplification of V_H_H from lymphocyte cDNA

Previous V_H_H cDNA libraries used for high-throughput sequencing or display approaches to nanobody selection have generally relied on primer sets complementary to the relatively conserved FR1 and FR4 framework regions of the V_H_H domain (10, 21, 22). However, in other studies, we have found surprising amounts of divergence in these framework regions (4, 14), and so sought to evaluate the V_H_H diversity that our existing primer sets were able to amplify.

Given the higher conservation outside of the V_H_H domain, we first performed long read PacBio sequencing of PCR products amplified from llama or alpaca lymphocyte cDNA by primers annealing to the flanking IgG leader (termed primer CALL001) and CH2 (CALL002) regions, as typically used in the first step of nested PCRs amplifying V_H_H (Fig. S2*A*) (21). V_H_H DNA was separated from longer V_H_ fragments (containing CH1) by agarose gel electrophoresis. We analyzed the first 18 bp of V_H_H domains from 6353 llama sequences and found that the top 20 most prevalent 5′ sequences came from only 47% of the population and contained up to five substitutions from the consensus sequence (Fig. S2*B*). In alpaca, 41% were covered by the top 20. This suggested that many divergent primers would be necessary to achieve representative amplification of the V_H_H population. Similar analysis of the 3′ end revealed higher conservation, with the top 20 sequences covering 92% of the population in both species, with three or fewer substitutions in this set.

To avoid potential PCR bias and assess sequences not amplified by CALL001/CALL002, we also surveyed the V_H_H sequence population by direct Illumina HiSeq sequencing of llama lymphocyte cDNA, resulting in 33 million paired-end reads. We first sought to determine whether the CALL001 primer annealing in the IgG leader sequence achieved adequate V_H_H coverage. To identify target IgG regions from sequencing reads, we searched for matches to the CALL001 sequence, allowing 20% mismatches (5 in 23 bp) to ensure less conserved sites were captured, without excessive off-target matching. The top ten most abundant sequences were selected, with less abundant sequences merged by allowing one mismatch to account for potential sequencing errors or point mutations (Fig. S2*C*). This top ten covered 87% of the identified sequences, including all those with IgG flanking regions consistent with genomic sequences (23). This analysis suggests that CALL001 indeed targets a highly conserved sequence in the IgG leader region suitable for comprehensive V_H_H amplification, with significantly higher conservation than alternatives targeting FR1.

While existing CH2 and FR4 3′ primers did appear to target well-conserved sequences, these present limitations for amplifying the V_H_H domain: first, use of FR4 primers necessitates a less efficient nested PCR strategy to separate V_H_ and V_H_H, as FR4 is conserved between these variants; and second, for purposes such as MS, it is useful to have sequence information into the flanking hinge region, as this allows better identification of CDR3 peptides extending into the hinge, and identifies the corresponding hinge variant (IgG2 or IgG3) for each sequence. While use of the downstream CALL002 CH2 primer would address these concerns, it would be too distant from the V_H_H region for typical library or sequencing approaches. We therefore aimed to design primers targeting the flanking V_H_H hinge regions.

**Figure 3:**
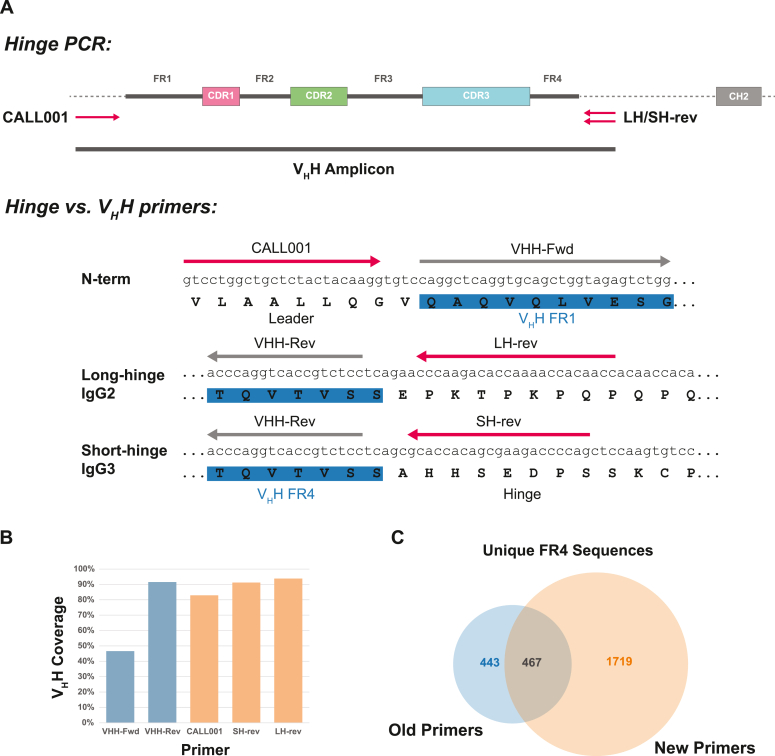
**Amplification of V**_**H**_**H fragments by high-coverage hinge-specific primers.***A*, schematic representation of hinge primers selected for higher coverage single-step PCR amplification of V_H_H regions. Forward primer CALL001 anneals in the leader region upstream of FR1, while reverse primers LH-rev and SH-rev anneal in the long-hinge or short-hinge region, respectively, downstream of FR4. In contrast, second step nested primers VHH-Fwd and VHH-Rev anneal in FR1 or FR4 regions. *B*, high-throughput sequencing of lymphocyte mRNA was used to determine what percentage of V_H_H-encoding transcripts could be recognized by primers used for traditional nested PCR (*blue*) or hinge-targeted PCR (*yellow*) by searching each primer against the total sequence database. *C*, Venn diagram comparing the number of unique FR4 sequences captured using each primer set, as determined by adjacent sequence in V_H_H transcript. New hinge primers correspond to a wider diversity of FR4 sequences. Incomplete sequencing coverage, FR4 mismatches from “old” V_H_H primers, and amplification of non-IgG sequences contribute to nonoverlapping sequences.

We identified candidate conserved sites in both long and short (IgG2 and IgG3) hinge regions immediately downstream of the V_H_H domain (Fig. 3*A*). Searching these candidate primer sites against cDNA sequencing reads and allowing up to 20% mismatches, we again merged matches into the top ten most abundant sequences. These accounted for 94% or 91% in long or short hinge samples, respectively (Figs. 3*B* and S2*C*). With less than one mismatch within each top ten set, these primer candidates are suitable for comprehensive amplification of V_H_H domains. Analysis of adjacent FR4 sequences also revealed significantly more diversity in this framework region when amplified using hinge primers, again indicative of the greater coverage achieved when targeting outside of the V_H_H domain (Fig. 3*C*).

The combination of leader and hinge primers allows comprehensive, single-step PCR amplification of V_H_H from cDNA. While this adds a further 27 bp upstream and 22/23 bp downstream of the V_H_H domain compared to FR1/FR4 primers, this did not interfere with sequencing or expression. These primers are also compatible with display library approaches from naïve or immunized camelids, and should similarly allow for increased V_H_H coverage of libraries generated for these methods by PCR amplification.

### Improved workflow for nanobody sequence identification

The Llama-Magic software that we previously used to correlate MS data to sequenced V_H_H transcripts was substantially updated to improve the confidence of nanobody candidate selection (14). This improved version is now available as Llama-Magic 2.0 (https://github.com/FenyoLab/Llama_Nanobodies). Merging of paired end reads was modified to account for new PCR amplicon composition, and *in silico* digestion can be performed with both trypsin and chymotrypsin, with peptides matched to the corresponding digested sample. Sequences are scored by the degree of peptide coverage with additional weight being given to CDR coverage. The resulting candidates are grouped by CDR3 sequence, where CDR3 groups are ranked by their top-scoring members. Then, sequences within CDR3 groups are ranked by high-throughput sequencing counts; given the inherent errors in DNA sequencing and PCR fidelity, higher count sequences are more likely to be from *bona fide* transcripts, and have indeed correlated more strongly with true antigen binders. Additionally, we have found that increasing Illumina sequencing coverage above ∼10 million reads does not significantly increase the quality of top nanobody matches, suggesting that at least in this pipeline, rarer clones are not an essential component of the captured high-affinity serum HCAbs.

Each peptide identified by mass spectrometry is assigned a “uniqueness score” for each CDR3 group to which it matches, calculated as 100 times the number of sequences in the CDR3 group that contain the peptide, divided by the number of sequences in the entire database that contain the peptide. A peptide’s uniqueness score is therefore the probability that it originated from an antibody with the given group’s CDR3. Selection of a candidate sequence requires at least one peptide with a high uniqueness score (>80). Typically, peptides containing the CDR3 are the most unique within a given V_H_H sequence. The MS/MS spectra of these peptides are examined to confirm fragmentation within any part of the CDR3 they may span. The same logic is applied to peptides that span parts of CDR1 and 2. The likelihood that a sequence will ultimately bind to the target antigen is increased by unique peptide coverage across its length.

Candidate lists generated from Llama-Magic 2.0 output were manually filtered with the following additional criteria. First, sequences that were identified in V_H_H samples purified against unrelated antigens were eliminated as likely arising from nonspecific binding, and sequences containing the GLEW motif at residues 49 to 52 (IMGT numbering) were also removed as likely VH contaminants (24). Candidates passing these filters were selected for gene synthesis, expression as nanobodies, and antigen-binding screening (14). With the increased stringency of this candidate identification and upstream sample preparation, the false discovery rate is substantially lower than previous results: the overall percentage of selected candidates with verified antigen binding increased from 57% (37/65) using our prior method (14) to 76% (69/91) in this report, and is routinely as high as 90% with other antigens (4).

### Nanobody repertoires obtained with our improved MS-based methodology

**Figure 4:**
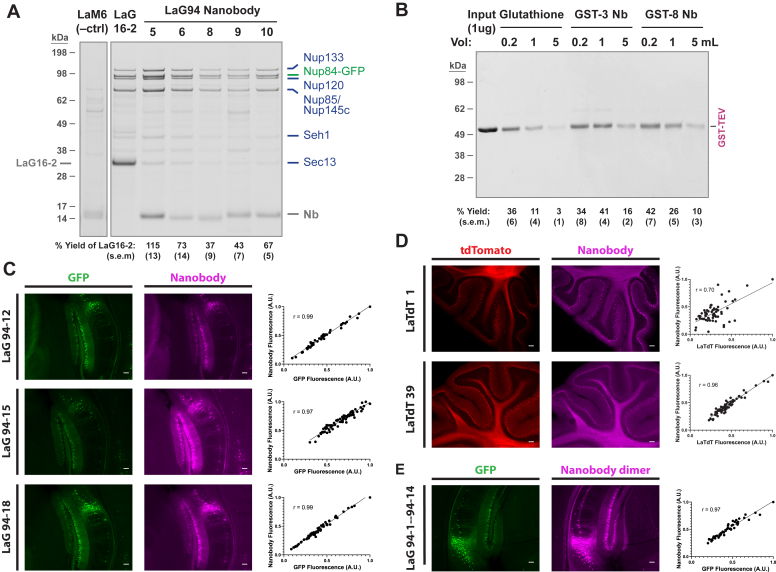
**GFP and tdTomato nanobody performance in tissue IF and affinity purifications.***A*, GFP nanobodies were conjugated to Dynabeads and used to affinity purify Nup84-GFP and its associated NPC subcomplex from yeast. An anti-mCherry nanobody (LaM6) was used as a negative control for nonspecific background, and the LaG16-2 nanobody heterodimer used as the highest affinity nanobody reagent previously reported (14). Complexes were eluted in SDS and analyzed by Coomassie-stained SDS-PAGE. Band densities of Nup133, Nup84-GFP, Nup120, and Nup85/Nup145c were quantified and normalized to the LaG16-2 lane (n = 3). *B*, Briefly, 1 μg of GST-TEV was diluted in 0.2 to 5 ml of *Escherichia coli* lysate and isolated with glutathione- or nanobody-coupled Sepharose, with SDS elutions analyzed by Coomassie-stained SDS-PAGE. Recovery of the GST-tagged protein was quantified by densitometry (n = 3). *C*, new GFP nanobodies were labeled with Alexa Fluor 568 and used to stain brain sections of Thy1-GFPM mice. Quantitative comparisons of relative GFP and nanobody fluorescence are plotted at *right*. Fluorescence was normalized to maximum fluorescence in each channel. Plots were fitted with simple linear regression, and Pearson correlation coefficients (r) are indicated. The scale bars represent 100 μm. *D*, tdTomato nanobodies were labeled with Alexa Fluor 647 and used to stain sagittal brain sections of ChAT-Cre::Ai14 mice, with tdTomato expressed in cholinergic neurons. Quantification is shown as in (*C*). The scale bars represent 100 μm. *E*, a GFP nanobody heterodimer was labeled with Alexa Fluor 647 and used for Thy1-GFPM brain section staining as in (*C*). The scale bars represent 100 μm. IF, immunofluorescence; NPC, nuclear pore complex; TEV, tobacco etch virus.

To validate the improvements made to our nanobody generation pipeline, we applied it to several target antigens of interest. GFP was chosen as a benchmark for comparison with previous studies, and remains of intense biomedical interest, with sustained demand for reagents of the highest possible affinity for a variety of applications. After GFP immunization of a single llama, we assessed the top 20 candidates from MS analysis of lymphocyte V_H_H and affinity purified serum, confirming binding of 16 by an *in vitro* binding screen. Surface plasmon resonance (SPR) was used to measure the binding kinetics and affinities for the 12 of these nanobodies that gave the best immunofluorescence microscopy signal (see below), all of which were found to bind strongly (K_D_s from 2.7 × 10^−8^ to <1.4 × 10^−13^ M) (Table 1 and Fig. S3). These nanobodies were also tested for affinity isolations after covalent conjugation to magnetic Dynabeads. In a representative purification of Nup84-GFP from yeast, the tested nanobodies isolated the associated Nup84 complex at comparable or better yield and purity to the best previously identified GFP nanobody dimer (Fig. 4*A*) (14).

**Table 1:** Summary of SPR binding kinetics data

Nanobody ID	k_on_ (1/s)	k_off_ (1/Ms)	K_D_ (M)	k_a_ sem	k_d_ sem	K_D_ sem	n
LaG94-1	1.1E+06	1.8E-05	1.6E-11	3.0E+05	2.3E-06	4.7E-12	5
LaG94-2	1.1E+05	3.0E-03	2.7E-08	1.4E+04	4.1E-04	4.8E-09	3
LaG94-3	1.1E+07	1.5E-03	1.3E-10	1.5E+06	2.5E-04	2.9E-11	3
LaG94-4	4.1E+05	1.5E-03	3.7E-09	4.5E+04	1.8E-04	5.9E-10	3
LaG94-5	5.6E+06	<5.0E-06	<8.9E-13	7.8E+05	n/a	1.2E-13	5
LaG94-6	5.2E+06	4.3E-04	8.3E-11	8.1E+05	5.9E-05	1.7E-11	3
LaG94-8	5.5E+06	1.2E-03	2.1E-10	4.3E+06	7.5E-04	2.1E-10	3
LaG94-9	4.5E+07	9.5E-04	2.1E-11	2.4E+07	3.7E-04	1.4E-11	3
LaG94-10	3.9E+06	3.5E-05	9.0E-12	1.1E+06	9.9E-06	3.6E-12	5
LaG94-12	1.3E+06	4.1E-04	3.0E-10	1.9E+05	5.3E-05	5.8E-11	3
LaG94-15	3.6E+07	<5.0E-06	<1.4E-13	2.2E+07	n/a	8.6E-14	3
LaG94-18	3.3E+05	3.1E-04	9.2E-10	9.2E+04	4.1E-05	2.8E-10	3
LaTdT-1	2.8E+05	<5.0E-06	<1.8E-11	6.0E+04	n/a	3.8E-12	2
LaTdT-2	1.3E+07	4.5E-05	3.5E-12	1.1E+06	1.4E-05	1.1E-12	2
LaTdT-8	1.3E+07	4.8E-04	3.7E-11	4.4E+06	1.1E-04	1.5E-11	4
LaTdT-9	2.1E+05	1.8E-05	8.4E-11	4.8E+04	1.0E-05	5.2E-11	4
LaTdT-10	7.9E+05	4.1E-05	5.2E-11	1.5E+05	1.4E-05	2.1E-11	4
LaTdT-38	1.7E+06	9.7E-05	5.7E-11	2.9E+05	1.8E-05	1.5E-11	4
LaTdT-39	1.1E+06	6.3E-06	5.9E-12	4.8E+04	1.0E-05	5.2E-11	3
LaTdT-44	1.1E+06	4.9E-05	4.4E-11	2.7E+05	1.2E-05	1.5E-11	2
LaTdT-45	4.7E+05	1.3E-05	2.8E-11	4.4E+03	2.8E-07	6.4E-13	2
GST-1	1.1E+06	9.2E-05	8.5E-11	1.2E+05	4.2E-06	1.0E-11	3
GST-2	1.2E+06	7.9E-05	6.4E-11	4.3E+04	2.1E-05	1.7E-11	2
GST-3	1.4E+06	4.8E-04	3.5E-10	6.0E+05	7.1E-05	1.6E-10	3
GST-4	4.0E+05	1.1E-04	2.6E-10	1.5E+05	3.5E-05	1.3E-10	3
GST-5	8.7E+05	1.1E-04	1.3E-10	2.3E+05	3.9E-05	5.7E-11	3
GST-6	4.1E+06	6.1E-04	1.5E-10	2.2E+05	3.8E-05	1.2E-11	2
GST-7	4.3E+06	7.6E-04	1.7E-10	7.8E+04	6.6E-05	1.6E-11	2
GST-8	2.4E+05	3.1E-05	1.3E-10	9.8E+04	1.0E-05	6.9E-11	2
LaMIgG-5	2.8E+05	1.2E-04	4.2E-10	1.4E+05	3.6E-06	2.1E-10	2
LaMIgG-8	1.3E+05	1.6E-04	1.2E-09	2.8E+04	1.7E-06	2.8E-10	2
LaMIgG-9	2.9E+05	1.8E-04	6.0E-10	3.3E+04	1.9E-05	9.3E-11	2
LaMIgG-14	2.2E+05	4.7E-05	2.1E-10	1.6E+04	8.6E-06	4.3E-11	2
LaRIgG-1^a^	8.9E+05	3.4E-03	3.6E-10	1.4E+04	7.1E-04	9.6E-11	2
	2.6E-03	2.6E-04		1.5E-04	2.9E-05		
LaRIgG-2	3.6E+05	<5.0E-06	<1.4E-11	1.5E+05	n/a	6.0E-12	4
LaRIgG-7	9.6E+05	5.1E-05	5.3E-11	1.2E+05	1.0E-06	6.6E-12	2
LaGIgG-4^a^	2.2E+06	1.1E-02	8.2E-10	1.9E+05	1.9E-03	3.6E-10	2
	1.8E-03	3.3E-04		2.7E-04	3.0E-05		
LaGIgG-12	3.2E+05	1.6E-04	4.9E-10	6.7E+04	2.6E-05	1.3E-10	3

a 2-state binding model.

Alongside GFP, we coimmunized the same animal with tdTomato, a highly divergent synthetic red fluorescent protein dimer derived from *Discosoma* sp (25). This is a widely used tag often applied in transgenic animals for immunohistochemistry (IHC) studies due to its favorable brightness, maturation, and folding properties (26, 27) and for which a high-affinity bait is desirable, *e.g.*, for complementary interactomic studies. We again identified 31 top candidates, 21 of which passed our initial binding screen. Nine were assessed by SPR, demonstrating consistently excellent affinity (K_D_s from 8.4 × 10^−11^ to 3.5 × 10^−12^ M) (Table 1 and Fig. S4).

As an additional target we selected GST, most commonly used as a fusion protein tag for recombinant protein expression and affinity purification (28). While immobilized glutathione is routinely used for GST isolation, anti-GST nanobodies provide complementary tools for isolation or detection of GST-tagged proteins, enabling new applications due to their flexibility as encodable, modifiable proteins (29). We generated eight high-affinity nanobodies against GST, verified by SPR (K_D_s from 3.5 × 10^−10^ to 6.4 × 10^−11^ M) (Table 1 and Fig. S5). As a benchmark for specificity and affinity, we compared these nanobodies to glutathione Sepharose in affinity isolations of a GST-tagged protein (GST-tobacco etch virus [TEV]) from *Escherichia coli* lysate. In increasing dilutions of the tagged protein, at least two nanobodies showed superior recovery of GST, with comparable high purity (Fig. 4*B*). With this high affinity, high specificity performance, these reagents are well suited to the efficient capture or detection of GST-tagged targets.

We further immunized three animals (one alpaca and two llamas) with IgG from mouse, rabbit, or goat. Given the ubiquity of antibodies generated in these species, new anti-IgG nanobodies continue to be valuable tools for use as single domain substitutes for traditional secondary antibodies. Our pipeline identified 14, 6, and 12 nanobodies against mouse, rabbit, and goat IgG respectively, from 16, 9, and 15 top candidates. SPR again demonstrated high affinities for nine tested IgG nanobodies (K_D_ from 1.2 × 10^−9^ M to <1.4 × 10^−11^) (Table 1 and Fig. S6).

### Nanobodies as optimized tools for immunofluorescence microscopy

Sensitive fluorescent detection of protein tags, particularly IHC in fixed cells or tissues, demands high-performance antibodies. Dye-labeled nanobodies offer particular advantages in this application. Their small size allows significantly faster penetration into samples, particularly important for volume imaging where antibody diffusion is a significant bottleneck (30, 31). This small size also contributes to more precise localization in high-resolution microscopy. Further, nanobodies bind at a single epitope, and thus potentially can have a 1:1 binding ratio that leads to a linear response in signal, an important factor in quantitative analysis.

To evaluate the effectiveness of our new nanobody repertoires in IHC, we screened fluorescently labeled nanobodies against brain sections from transgenic mice expressing GFP or tdTomato in the brain (Thy1-GFP or ChAT-Cre/Ai14 tdTomato reporter). Out of 16 GFP nanobodies tested, nine demonstrated the strongest fluorescence correlating to GFP signal (Figs. 4*C* and S7). Similarly, five tdTomato nanobodies proved the most effective out of 20 screened (Figs. 4*D* and S8). Importantly, the fluorescent signal intensity of these GFP nanobodies has a strong linear correlation to GFP itself (Pearson r ≥ 0.92). While this correlation, and overall signal to noise performance, is somewhat weaker for most tdTomato candidates, LaTdT-39 performs at a similarly high level (r = 0.96).

### Epitope screening and oligomerization of GFP nanobodies for immunohistochemistry

To further improve the sensitivity and specificity of nanobodies in this application, we sought to engineer bivalent, heterodimeric constructs with increased avidity. To determine the optimal combinations of nanobodies for this purpose, we first binned epitopes by complementary staining of nanobody pairs in IHC. We have previously established the binding sites of reported GFP nanobodies, which fell into three nonoverlapping epitope groups (14). Representative nanobodies binding these three epitopes (LaG16, LaG41, and LaG2) were used to screen new GFP nanobodies by competitive binding in IF. These controls were labeled with a different fluorophore, and used to prestain a GFP tissue sample to saturation before probing with the new nanobody set (Figs. S9–S11) (14). When we observed simultaneous fluorescent signal from both nanobodies, the pair was judged to be complementary, binding distinct epitopes. In addition to providing biochemical epitope information, this approach revealed which pairs of nanobodies could achieve complementary, simultaneous binding in the IHC environment of practical interest. Most of the tested anti-GFP nanobodies did in fact show complementation with the three representative nanobodies tested. Three (LaG94-1, LaG94-10, and LaG94-12) appear to overlap with LaG41’s epitope, and in fact outcompete LaG41 during costaining. To further classify the remaining nanobodies, we performed a similar complementation screen between LaG94-10, 14, 15, and 18 (Fig. S12). All four were able to complement each other, indicating distinct epitopes.

With the numerous complementary nanobody pairs revealed by these experiments, we generated a series of dimeric constructs. These were again assessed in IHC of Thy1-GFP brain sections, and while more variance was found in signal to noise and linearity performance, at least two dimers, LaG94-1—LaG94-14 and LaG94-14—LaG94-12, showed significantly improved specificity and signal strength while retaining linear staining (Figs. 4*E* and S13).

### Anti-IgG nanobodies as improved secondary antibodies

**Figure 5:**
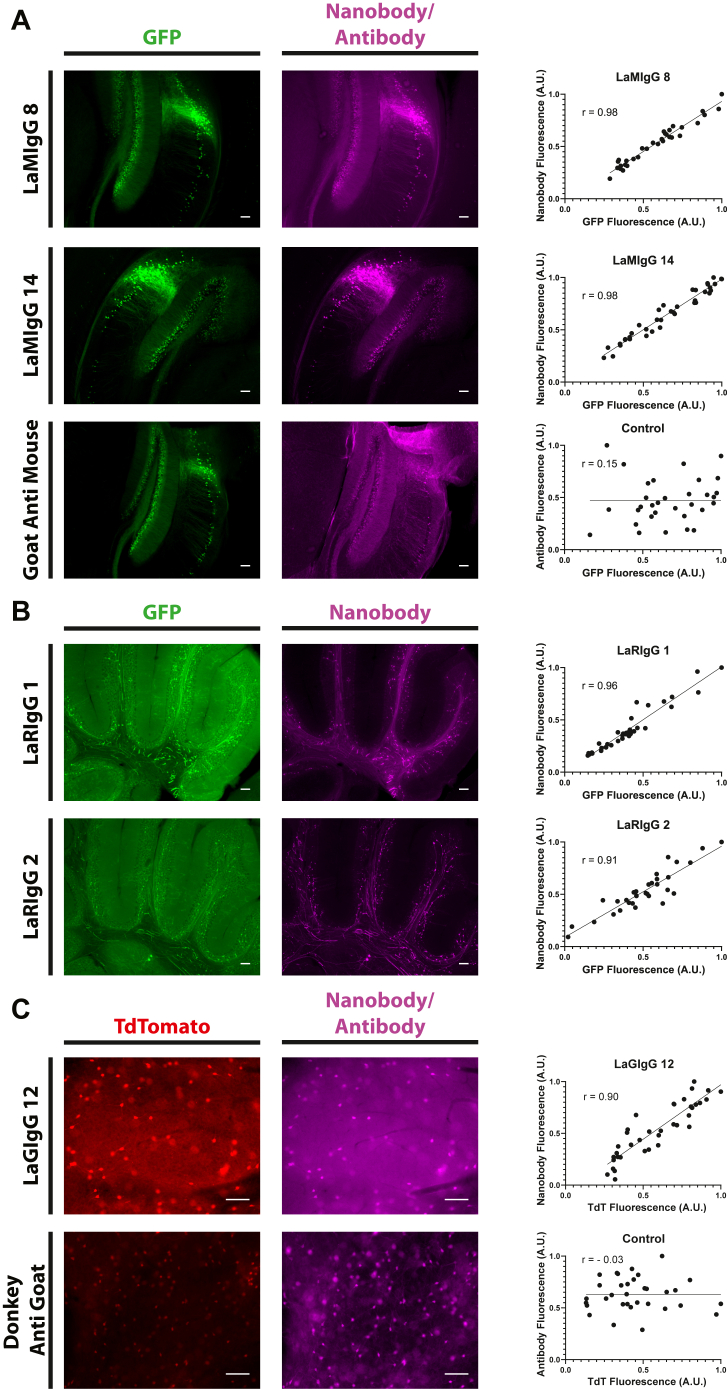
**IgG nanobodies as linear secondary detectors in tissue IF.** Nanobodies against mouse IgG (*A*), rabbit IgG (*B*), or goat IgG (*C*) were fluorescently labeled with Alexa Fluor 568 (mouse and rabbit) or Alexa Fluor 647 (goat) and used to detect anti-GFP or anti-tdTomato primary antibodies in Thy1-GFPM (*A* and *B*) or somatostatin-TdTomato (*C*) mouse brain sections. Standard monoclonal anti-mouse and polyclonal anti-goat secondaries were also tested. Quantitative comparisons of relative GFP or tdTomato and nanobody/secondary fluorescence are plotted at *right*. Fluorescence was normalized to maximum fluorescence in each channel. Plots were fitted with simple linear regression, and Pearson correlation coefficients (r) are indicated. The scale bars represent 100 μm. IF, immunofluorescence; IgG, immunoglobulin G.

Mouse, rabbit, and goat IgG nanobodies were similarly tested against Thy1-GFP brain sections first stained with anti-GFP monoclonal antibodies from the corresponding species (Figs. 5 and S14). Strong, linear staining was found with several of these nanobodies: 5 anti-mouse, 3 anti-rabbit, and 1 anti-goat. The signal specificity for these top performers was comparable to a standard mouse and goat polyclonal secondary antibody; however, the linear correlation of signal was far higher (Pearson r = 0.76–0.98 *versus* r = −0.03–0.15). These monoclonal nanobody agents are thus significantly better suited to IHC applications where antigen localization must be quantitatively compared.

We further categorized the species and isotype specificity of the IgG nanobodies effective in IHC by immunoblotting (Fig. S15). Most were highly specific for IgG from their target species, with the exceptions of goat G-4 and G-12 (sheep cross-reactivity), rabbit R-2 (human cross-reactivity), and mouse M-5, M-8, and M-14 (rat cross-reactivity). Mouse nanobody M-9 bound all mouse IgG isotypes other than IgG1 λ, while others specifically targeted IgG2b. The tested nanobodies targeted the Fc domain, other than rabbit R-1 and the mouse nanobodies, which bound F(ab’)2.

## Discussion

For any nanobody application, rapid and efficient identification of high-affinity clones is a critical first step. We previously demonstrated a nanobody pipeline making use of MS and next-generation DNA sequencing, with advantages in rapid generation of large repertoires, and identification of ultra-high-affinity candidates from high-stringency selection. We have now made a variety of improvements to this pipeline, significantly increasing coverage of V_H_H libraries from immunized animals and improving the quality of MS samples, including in the cases of highly conserved or weakly reactive immunogens. This pipeline can generate large numbers of nanobody candidates as needed, and we have for example validated well over 100 high-affinity nanobodies from a single antigen (4).

Using these improved methods, we have identified new repertoires of nanobodies against a panel of protein antigens with immediate value in research applications: GFP, tdTomato, GST, and rabbit, mouse, and goat IgG. These repertoires build significantly on previously reported nanobodies against these or related targets (14, 29, 32, 33), *i.e.*, with significantly higher affinity, increased repertoire size covering multiple epitopes, and against new and widely used antigens like tdTomato and goat IgG. These nanobodies have affinities into the picomolar range, and demonstrate strong activity in both affinity capture and IF applications. We have increased affinity and performance even further by identifying epitope bins and engineering dimeric nanobodies, focusing on anti-GFP reagents. Nanobodies have a variety of intrinsic advantages as affinity reagents, particularly in IF applications. The 10-fold smaller size of nanobodies compared to standard IgG secondaries provides for faster diffusion, allowing for more efficient staining of thicker sections or whole-mount samples (30, 31). This small size is a further advantage for super-resolution microscopy, with the significantly closer proximity of the covalently conjugated fluorophore allowing more precise localization of the target antigen (34, 35).

## Experimental procedures

### Llama immunizations and bleeds

Llama immunizations were performed as previously described (14). Briefly, naïve animals were immunized with an antigen mixture containing 200 μg to 1 mg of each antigen protein, emulsified with one volume of complete Freund’s adjuvant. Three subsequent boosts were performed at 21 days intervals, using incomplete Freund’s adjuvant. Animals previously immunized (>6 months prior) instead received both primary and boost immunizations with incomplete Freund’s adjuvant. Production serum bleeds and bone marrow aspirates were obtained 10 days after the final boost.

### V_H_H sequencing

Lymphocytes were isolated from bone marrow aspirates using a Ficoll-Paque gradient separation (Cytiva). RNA was isolated using TRIzol (Thermo Fisher Scientific), and cDNA was generated using SuperScript IV reverse transcriptase with oligo(dT) primers (Thermo Fisher Scientific). V_H_H domains were then PCR amplified using Deep Vent polymerase (NEB). Nested PCRs were performed as previously described using CALL001/CALL002 primers, followed by MiSeq-VHH-forward and MiSeq-VHH-reverse primers (14). Single-step PCRs using hinge-specific primers were performed with 6N-CALL001/6N-CALL001b (5′-NNNNNN GTCCTGGCTGCTCTT[C/T]TACAAGG-3′) forward primers and a 1:1 mixture of 6N-SH-rev (5′-NNNNNN CTGGGGTCTTCGCTGTGGTGC-3′) and 6N-LH-rev (5′-NNNNNN GTGGTTGTGGTTTTGGTGTCTTGGG-3′) reverse primers. PCR products were gel-purified, ligated to Illumina adapters for library preparation, and sequenced by MiSeq with 2 × 300-bp paired end reads.

CALL001 and CALL002 primers were used to PCR amplify IgG fragments from llama and alpaca bone marrow lymphocyte cDNA, which were gel-purified and prepared using a 20kb SMRTBell library prep kit (PacBio) for sequencing on a PacBio RS II system. For sequencing of llama lymphocyte transcripts, mRNA was enriched from total RNA by oligo(dT) capture, and libraries prepared using the TruSeq Stranded mRNA Sample Preparation Kit (Illumina). NextSeq Mid Output 2 × 75-bp paired end sequencing was carried out on an Illumina NextSeq 500 instrument.

For *in silico* protease digests, sequences from translated databases (see Llama-Magic 2.0 below) were numbered using ANARCI software with IMGT numbering (https://opig.stats.ox.ac.uk/webapps/sabdab-sabpred/sabpred/anarci/) (36). In silico digestion was performed using Pyteomics with default Expasy cleavage rules for trypsin, chymotrypsin high specificity, and chymotrypsin low specificity, and no missed cleavages (https://pyteomics.readthedocs.io/en/latest/_modules/pyteomics/parser.html) (37, 38).

### HCAb preparation

Heavy-chain only variant IgG (HCAb) was first enriched from crude serum using Protein A and Protein G as previously described (1, 14). Protein M was separately expressed and purified in bacteria, and conjugated to CNBr-activated Sepharose 4 Fast Flow (Cytiva) at a ratio of 1 mg Protein M to 50 mg Sepharose (final coupling density of approximately 5 mg per ml of swelled Sepharose). Residual light-chain containing IgG was then depleted from Protein A/G-fractionated HCAb by incubating with PrM-Sepharose (50 μl Sepharose bed volume per 1 mg IgG) for 30 min at room temperature and collecting flow-through.

### HCAb affinity capture and IdeS digestion

In total, 10 mg of purified HCAb was affinity purified over 100 μl of CNBr-Sepharose coupled to the corresponding antigen protein at 1 mg/ml. The resin was washed sequentially with PBS-500 (500 mM NaCl final concentration), PBS + 0.5% Triton, 2 to 3 M MgCl_2_/10 mM Tris pH 7.5, and PBS. The resin was then resuspended in 200 μl PBS and digested with 400 units of IdeS (FabRICATOR, Genovis) for 3.5 h, mixing at 37 °C. Comparative samples were digested with papain as previously described (14), or eluted in lithium dodecyl sulfate (LDS) without digestion. Bound protein was eluted by incubating 10 min at 72 °C with 1.2× LDS loading buffer (Thermo Fisher Scientific). Samples were reduced, alkylated with iodoacetamide, and separated by SDS-PAGE. V_H_H bands at 15 to 25 kDa were excised and prepared for MS analysis.

### Mass spectrometry

Trypsin (Promega) or chymotrypsin (Roche) solution was added to diced, destained, and dehydrated gel pieces at ∼1:4 enzyme to substrate mass ratios in 55 μl digestion buffer (trypsin: 50 mM ammonium bicarbonate; chymotrypsin: 100 mM Tris pH 8.0, and 2 mM CaCl2). In the case of trypsin, enzyme was supplied in two additions of equal mass each. After the first addition, the samples were incubated at 37 °C for 4 h. After the second addition, the samples were incubated at 37 °C overnight. For chymotrypsin, one of the incubation was performed for 1.5 h at 37 °C. Supernatants were removed from gel pieces and transferred to new tubes. To extract peptides, 100 μl of a 1.7% v/v formic acid, 70% v/v acetonitrile, and 0.1% v/v trifluoroacetic acid solution were added to the gel pieces, and tubes were shaken at 37 °C for 1 h. Supernatant was removed from gel pieces and combined with previous supernatant. Sequential extractions were performed similarly with 100 mM triethylammonium bicarbonate and acetonitrile. The pooled supernatants were evaporated in a speedvac until dry. Peptides were resuspended in 5% v/v formic acid, 0.1% v/v trifluoroacetic acid, and cleaned on StageTips (39).

Samples were analyzed with a nano-LC 1200 (Thermo Fisher Scientific) using an EASYspray PepMap RSLC C18 3 micron, 100 Å, 75 μm by 15 cm column coupled to an Orbitrap Fusion Lumos Tribrid mass spectrometer (Thermo Fisher Scientific). The instrument was operated in data-dependent mode, and top intensity ions were fragmented by collision-induced dissociation (collision energy 35). Ions with charge states 2 to 6 were selected for fragmentation. The quadrupole isolation window was 1.4, and the MS/MS used a maximum injection time of 250 ms with 1 microscan.

### Llama-Magic 2.0

The Llama-Magic github repository is located at https://github.com/FenyoLab/Llama_Nanobodies. It includes all code, as well as detailed instructions for running the custom scripts for database preparation and for setting up and using the Llama-Magic web tool. Detailed methods are included in supporting information.

In brief, to identify candidate nanobody sequences, a custom database of *in silico* translated protein sequences is created from the DNA sequencing reads. After merging the read pairs, the correct ORF is selected by determining the position of the PCR primers near the start and end of each sequence. The protein sequence database is then *in silico* digested with both trypsin and chymotrypsin and lists of unique tryptic and chymotryptic peptides are saved as FASTA files. The tandem MS data are searched against these peptide lists and identified peptides from both tryptic and chymotryptic digests are mapped back to the protein sequences. In order to determine MS coverage over the CDR regions, we implement a custom algorithm that searches for specific conserved amino acid motifs proximate to CDR start and end sites. Each sequence is scored and ranked based on coverage over the CDR regions, with CDR3 coverage carrying the most weight. Sequences with similar CDR3 regions are grouped together in the output. Each identified peptide is given a uniqueness score that assesses its uniqueness within the CDR3 group relative to how often it is found in the overall sequence database.

### Protein preparation

GFP, tdTomato, and GST-TEV protease were produced in bacteria according to standard protocols. IgG proteins were purchased from Jackson Immunoresearch. Nanobodies were expressed in ArcticExpress (DE3) cells (Agilent Technologies) with a pelB leader sequence and C-term 6xHis tag, and purified from periplasm as previously described (14). For IF experiments, nanobodies were labeled with amine-reactive dyes as follows. First, 1.2 μl of 1 M NaHCO_3_ was added to 10 μl of 1.25 mg/ml nanobody. For Alexa Fluor 568 conjugation, 1 μl of 6 mg/ml Alexa Fluor 568 NHS Ester (Thermo Fisher Scientific, A20003) was added. For Alexa Fluor 647 conjugation 1 μl pf 8 mg/ml Alexa Fluor 647 NHS Ester (Thermo Fisher Scientific, A20006) was added. Samples were incubated at 37 °C for 1 h. Reaction was quenched by addition of PBS with 0.2% Triton X-100 and 10 μg/ml heparin (PTxH) by diluting to 10 μg/ml.

### Binding kinetics measurements by SPR

GFP nanobody SPR was performed on a ProteOn XPR36 (Bio-Rad) or Biacore 8K (Cytiva) instrument, in both cases essentially as previously described (4, 14). The Biacore 8K was used for all other antigens. For ProteOn measurements, GFP was immobilized on a GLC sensor chip (Bio-Rad) at 5 μg/ml in 10 mM sodium acetate pH 5.0 after EDC/NHS activation. Parallel binding measurements were performed at four or five nanobody concentrations run at 50 μl/min, with an association time of 180 s and a dissociation time of 960 s. Data processing and analysis were done with ProteOn Manager software (https://www.bio-rad.com/en-us/product/proteon-manager-software), fitting to a Langmuir binding model.

For Biacore measurements, antigens were immobilized at 1 μg/ml (tdTomato) or 5 μg/ml (GFP, GST, and IgGs) in 10 mM sodium acetate pH 5.5 on Series S CM5 sensor chips, using an EDC/NHS amine coupling kit (Cytiva) according to the manufacturer’s guidelines. Nanobodies were injected at 30 μl/min in single-cycle kinetics experiments at concentrations of 0.1, 0.3, 1, 3, and 10 nM, with an association time of 180 s, and a dissociation time of 1800 to 7200 s, depending on observed off-rate. Data were analyzed using Biacore software (https://www.cytivalifesciences.com/en/us/shop/protein-analysis/spr-label-free-analysis/spr-software-and-extensions/biacore-insight-evaluation-software-p-23528), fitting a Langmuir 1:1 binding model to sensorgrams to calculate kinetic parameters. RIgG-1 and GIgG-4 nanobodies were instead fit to a two-step binding model.

All binding experiments were conducted in a buffer of 20 mM Na-Hepes pH 7.4, 150 mM NaCl, 0.05% Tween 20. Sensor surfaces were regenerated between measurements with 10 mM glycine-HCl pH 3.0 (IgGs) or 3.5 M MgCl_2_ in 10 mM Tris–HCl pH 7.5.

### Affinity purifications

For yeast Nup84-GFP isolations, recombinant nanobodies were conjugated to epoxy-activated M-270 magnetic Dynabeads (Thermo Fisher Scientific) as previously described (14). Briefly, 10 μg nanobody protein was used per 1 mg of Dynabeads, with conjugations carried out in 0.1 M sodium phosphate, pH 8.0 and 1 M ammonium sulfate, incubating for 18 to 20 h rotating at 30 °C. Affinity isolations were carried out as previously described, using binding buffer consisting of 20 mM Hepes, pH 7.4, 500 mM NaCl, 2 mM MgCl_2_, 0.1% CHAPS, 0.1 M PMSF, and 3 μg/ml pepstatin A (14, 40). For each experiment, 25 μl of bead slurry was used with 0.25 g of yeast cell powder resuspended in 2.25 ml buffer.

For GST isolations, nanobodies were conjugated to NHS-activated Sepharose (Cytiva) according to the manufacturer’s instructions. Recombinant GST-fused TEV protease was diluted in 0.5 mg/ml lysate prepared from BL21 DE3 bacterial cells, which were resuspended in 20 mM sodium phosphate, pH 7.4, 350 mM NaCl, 0.1% Tween 20, 0.1 M PMSF, 3 μg/ml pepstatin A, and lysed by microfluidizer. A 10 μl bed volume of nanobody-Sepharose, or commercial glutathione Sepharose (GenScript) was incubated for 30 min with each sample, and then washed twice with 20 mM sodium phosphate, pH 7.4, 350 mM NaCl, and 0.1% Tween 20. Bound protein was eluted with LDS at 72 °C and analyzed by Coomassie-stained SDS-PAGE.

### Immunoblots

For nanobody dot blots, 500 ng IgG (250 ng for subtype-specific IgG) was spotted in 1 μl volume on nitrocellulose strips. After drying, membranes were blocked with 5% milk/PBS for 30 min, and then probed with Alexa Fluor 488-labeled nanobodies at 300 nM in 5% milk/PBS for 30 min. After two 10 min washes in PBS, blots were imaged with a ImageQuant LAS 4000 fluorescent imager using Cy3 filters (Cytiva).

### Immunohistochemistry of brain sections

Adult female mice were anesthetized with isoflurane and fixed with intracardiac perfusion of 4% paraformaldehyde in PBS. Samples were postfixed in 4% paraformaldehyde in PBS at 4 °C overnight. Subsequently, 100 μm sagittal sections were cut by vibratome from agar embedded brains.

Sections were washed twice with PTxH for 5 min and then 30 min. Nanobody was added at a concentration of 1 μg/ml for 5 h. Samples were then washed with PTxH for 1, 5, and 30 min followed by two 5-min washes in PBS. Samples were then mounted. For complementation experiments, following the 1, 5, and 30 min PTxH washes, the second nanobody was added at a concentration of 1 μg/ml for 5 h. Samples were then washed again in PTxH for 1, 5, and 30 min followed by two 5 min washes in PBS and mounting. For experiments using a nanobody secondary, primary antibodies were left on the section overnight. For LaMIgGs, a mouse anti-GFP (19C8, Memorial Sloan Kettering Monoclonal Antibody Facility) (41) at 1:500 was used as the primary antibody; for LaRIgGs, a polyclonal rabbit anti-GFP (abcam, ab290) at 1:500 was used; for the LaGIgGs, a polyclonal goat anti-mCherry (Scigen, Cat # AB0081-500) at 1:1000 was used. For LaMIgG the control secondary was 1:500 Goat anti-Mouse conjugated to Alexa Fluor 568 (Invitrogen). For LaGIgG control secondary was 1:500 Donkey anti Goat conjugated to Alexa Fluor 647 (Invitrogen). LaMIgG, LaGIgG, LaRIgG, and control secondaries were left on sections for 4 h.

### Imaging and image analysis

Images were acquired on a Nikon eclipse 90i upright fluorescent microscope, using either a 4× or 10× objective. Images were processed in ImageJ (https://imagej.net/). For Pearson’s Analysis the fluorescence of at least 40 cell somas was quantified. For each section, the values were normalized to the maximum fluorescence value in that channel. Values were plotted in GraphPad Prism (https://www.graphpad.com/) to calculate the Pearson correlation coefficient (r).

### Animal husbandry

All mouse care was performed in compliance with the protocols approved by the Institutional Care and Use Committee (IACUC) of The Rockefeller University. Llama care was performed at Capralogics, Inc according to protocols approved by their IACUC. The Thy1-GFPM, Ai14 (Rosa26^lox-stop-lox-tdTomato^), and ChAT-cre lines were acquired from the Jackson Laboratory.

## Data availability

Mass spectrometry datasets are deposited in the Zenodo repository, https://doi.org/10.5281/zenodo.10805358. Nanobody sequences are provided in supporting information (Table S1).

## Supporting information

This article contains supporting information (42, 43, 44).

## Conflict of interest

The authors declare that they have no conflicts of interest with the contents of this article.
