# Supplementary material for: A new generation of nanobody research tools using improved mass spectrometry-based discovery methods
Source: J Biol Chem. 2024 Aug 2;300(9):107623. doi: 10.1016/j.jbc.2024.107623 (PMC11401214; doi:10.1016/j.jbc.2024.107623)
Supplement: Supplemental Figures S1–S14 and Table S1 [file mmc1.pdf]

Supplementary Information

Fig S1. Efficacy and specificity of IdeS vs papain proteases for Fc cleavage, trypsin vs chymotrypsin digestion for MS, and gel slicing for MS. (A) 10 µg of representative proteins were digested with the indicated amount of papain or 67 Units of IdeS for 1 hour at 37°C, then analyzed by SDS-PAGE. (B) The serum HCAb fraction from an immunized llama was affinity purified on GFP Sepharose, then digested with papain or IdeS, or left undigested. Remaining resin-bound V_HH or HCAb was eluted in LDS and separated by SDS-PAGE before trypsin digest and LC-MS/MS analysis. Llama-Magic was used to generate nanobody candidate lists for each sample, which were then searched for GFP nanobody sequences previously identified from this serum. The positions of identified nanobodies in the total candidate lists are graphed. (C) Trypsin and chymotrypsin digestions of purified V_HH bands were analyzed by MS, and data searched either separately or together for nanobody identification. For each of the three searches, a list of fully unique matching sequences was generated. This list was filtered so that each CDR3 group is represented by the member with the largest number of DNA sequencing counts. This filtered list was sorted from high to low MS coverage. The number of identified CDR3 groups is plotted against the MS coverage percentage of the corresponding CDR3 region or total V_HH sequence. (D) V_HH purified against SARS-CoV-2 spike was separated by SDS-PAGE and the band sliced into 5 pieces analyzed separately by MS, or treated as a single band ("Non-sliced"). The cumulative count of unique PSMs over the 5 slices ("Slice 1-5") was significantly higher than the unsliced control. For two example V_HH sequences, relative intensities of three peptides covering CDR1, CDR2, and CDR3 were compared across the 5 slices. Differing enrichment patterns are seen consistently across the 3 peptides measured for each nanobody, assisting matching of CDR peptides to a single VHH sequence.

Fig S2. Comparing V_HH coverage of nested PCR and single-step hinge PCR. (A) Schematic comparison of traditional nested PCR and single-step hinge PCR protocols. (B) CALL001/CALL002 PCR amplicons from llama or alpaca lymphocyte cDNA were sequenced by PacBio, and the most abundant V_HH N-term and C-term 18 bp sequences were identified to assess potential priming locations. Percentage of the total analyzed population is indicated for each 18bp sequence. (C) Hinge PCR primers were searched against lymphocyte mRNA sequences to estimate relative coverage. The top 10 complementary sequence hits are shown, with percentage of total matches. Exact primer matches are indicated in blue, and mismatched nucleotides labeled in red. SH-rev matches also included off-target non-IgG sequences, indicated by asterisks.

Figure S3. SPR sensorgrams for GFP nanobodies. Representative sensorgrams from SPR binding experiments of nanobody analytes flowed over an immobilized GFP ligand are shown. Curves are from parallel binding kinetics experiments (LaG94-2, LaG94-4, LaG94-6, LaG94-10, LaG94-12, and LaG94-18) on a ProteOn instrument, or single-cycle kinetics experiments on a Biacore 8K instrument (injected nanobody concentrations = 0.1, 0.3, 1, 3, and 10 nM). Curves from a Langmuir 1:1 binding model were fitted.

Figure S4. SPR sensorgrams for tdTomato nanobodies. Representative sensorgrams from SPR binding experiments of nanobody analytes flowed over an immobilized tdTomato ligand are shown. Curves are from single-cycle kinetics experiments on a Biacore 8K instrument (injected nanobody concentrations = 0.1, 0.3, 1, 3, and 10 nM). Curves from a Langmuir 1:1 binding model were fitted.

Figure S5. SPR sensorgrams for GST nanobodies. Representative sensorgrams from SPR binding experiments of nanobody analytes flowed over immobilized GST-TEV are shown. Curves are from single-cycle kinetics experiments on a Biacore 8K instrument (injected nanobody concentrations = 0.1, 0.3, 1, 3, and 10 nM). Curves from a Langmuir 1:1 binding model were fitted.

Figure S6. SPR sensorgrams for IgG nanobodies. Representative sensorgrams from SPR binding experiments of nanobody analytes flowed over immobilized goat, mouse, or rabbit IgG ligands are shown. Curves are from single-cycle kinetics experiments on a Biacore 8K instrument (injected nanobody concentrations = 0.1, 0.3, 1, 3, and 10 nM). Curve fits are from a Langmuir 1:1 binding model, or a two-step binding model (asterisks).

Figure S7. Screening GFP nanobodies by immunofluorescence. (A) Schematic of nanobody conjugation and staining procedure. Nanobodies were labeled with Alexa Fluor 568 and used to stain brain sections of Thy1-GFPM mice. (B) Fluorescent imaging was performed on GFP or nanobody-AF568 signal. (C) Quantitative comparisons of relative GFP and nanobody fluorescence. Fluorescence was normalized to maximum fluorescence in each channel. Plots were fitted with simple linear regression, and Pearson correlation coefficients (r) are indicated. Scale bars are 100 µm. Micrographs and quantification panels for LaG94-12, LaG94-15, and LaG94-18 are reproduced here from Fig. 4C to aid in comparison between samples.

Figure S8. Screening tdTomato nanobodies by immunofluorescence. (A) Schematic of nanobody conjugation and staining procedure. Nanobodies were labeled with Alexa Fluor 647 and used to stain sagittal brain sections of ChAT-Cre::Ai14 mice, with tdTomato expressed in cholinergic neurons. (B) Fluorescent imaging was performed on tdTomato or nanobody-AF647 signal. (C) Quantitative comparison of relative tdTomato and nanobody fluorescence. Fluorescence was normalized to maximum fluorescence in each channel. Plots were fitted with simple linear regression, and Pearson correlation coefficients (r) are indicated. Scale bars are 100 µm. Micrographs and quantification panels for LaTdT-1 and LaTdT-39 are reproduced here from Fig. 4D to aid in comparison between samples.

Figure S9. Complementation of GFP nanobodies by immunofluorescence (LaG2). (A) Schematic of nanobody complementation IF procedure. Nanobodies were labeled with Alexa Fluor 568 (LaG2) or 647 (Complement) and used to stain brain sections of Thy1-GFPM mice in sequence. (B) Fluorescent imaging was performed on GFP, nanobody-AF568 (first nanobody, LaG2), or nanobody-AF647 signal (second nanobody, indicated at left). Scale bars are 100 µm.

Figure S10. Complementation of GFP nanobodies by immunofluorescence (LaG16). Nanobodies were labeled with Alexa Fluor 568 (LaG16) or 647 (Complement) and used to stain brain sections of Thy1-GFPM mice in sequence. Fluorescent imaging was performed on GFP, nanobody-AF568 (first nanobody, LaG16), or nanobody-AF647 signal (second nanobody, indicated at left). Scale bars are 100 µm.

Figure S11. Complementation of GFP nanobodies by immunofluorescence (LaG41). Nanobodies were labeled with Alexa Fluor 568 (LaG41) or 647 (Complement) and used to stain brain sections of Thy1-GFPM mice in sequence. Fluorescent imaging was performed on GFP, nanobody-AF568 (first nanobody, LaG41), or nanobody-AF647 signal (second nanobody, indicated at left). Scale bars are 100 µm.

Figure S12. Complementation of GFP nanobodies by immunofluorescence (LaG94-10,14,15). Nanobodies were labeled with Alexa Fluor 568 or 647 and used to stain brain sections of Thy1-GFPM mice in sequence. Sections were first stained with (A) LaG94-10, (B) LaG94-14, or (C) LaG94-15. Fluorescent imaging was performed on GFP, nanobody-AF568 (first nanobody), or nanobody-AF647 (second nanobody, indicated at left) signal. Scale bars are 100 μm.

Figure S13. Screening GFP nanobody dimers by immunofluorescence. GFP nanobody dimers were labeled with Alexa Fluor 647 and used to stain brain sections of Thy1-GFPM mice. (A) Fluorescent imaging was performed on GFP or nanobody-AF647 signal. (B) Quantitative comparisons of relative GFP and nanobody fluorescence. Fluorescence was normalized to maximum fluorescence in each channel. Plots were fitted with simple linear regression, and Pearson correlation coefficients (r) are indicated. Scale bars are 100 μm. Micrographs and quantification panels for LaG 94-1--94-14 are reproduced here from Fig. 4E to aid in comparison between samples.

Figure S14. Screening IgG nanobodies by immunofluorescence. (A) Schematic of nanobody conjugation and staining procedure. IgG nanobodies were labeled with Alexa Fluor 568 or 647 and used to stain brain sections of Thy1-GFPM probed with an anti-GFP antibody of the corresponding species (mouse or rabbit). For goat IgG nanobodies, somatostatin tdTomato mouse brain sections were stained with goat anti-tdTomato. Fluorescent imaging was performed on GFP/tdTomato or nanobody signal using (B) mouse IgG nanobodies, (C) rabbit IgG nanobodies, or (D) goat IgG nanobodies. Traditional rabbit or goat secondaries were also used as controls. Quantitative comparisons of relative GFP or tdTomato and nanobody/secondary fluorescence are plotted at right. Fluorescence was normalized to maximum fluorescence in each channel. Plots were fitted with simple linear regression, and Pearson correlation coefficients (r) are indicated. Scale bars are 100 μm. Micrographs and quantification panels for LaMIgG-8, LaMIgG-14, and Goat Anti Mouse are reproduced from Fig. 5A in (B); LaRIgG-1 and LaRIgG-2 from Fig. 5B in (C); and LaGIgG-12 and Donkey Anti Goat from Fig. 5C in (D), to aid in comparison between samples.

Figure S15. Species and isotype specificity of IgG nanobodies. Nanobodies were labeled with Alexa Fluor 488 and used to probe immobilized IgG of the indicated species, fragment, and/or isotype. An anti-GFP nanobody (LaG) was used as a negative control.

Figure S1

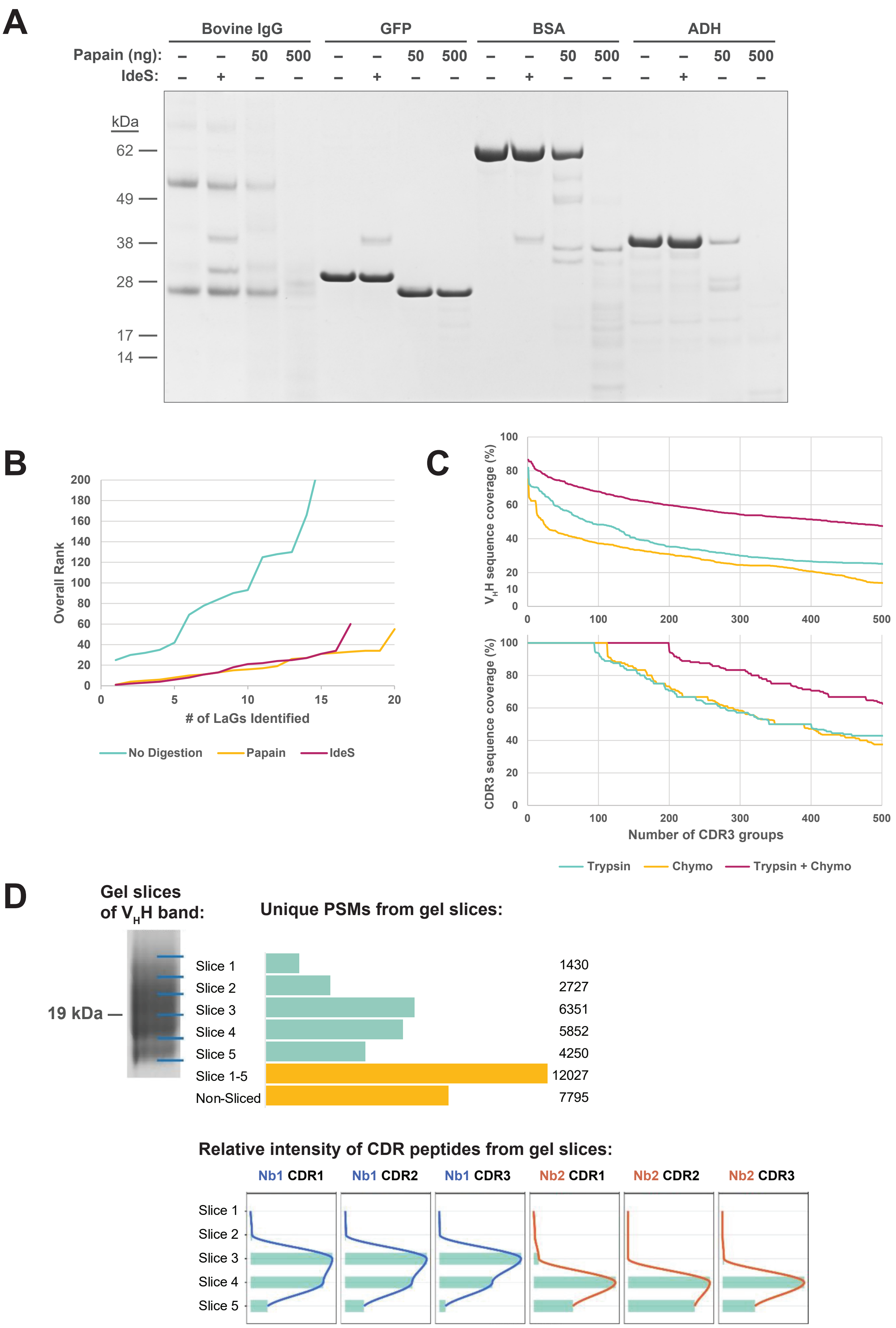
A
Bovine IgG
GFP
BSA
ADH
Papain (ng): – – 50 500 – – 50 500 – – 50 500 – – 50 500
IdeS: – + – – – + – – – + – – – + – –
kDa
62
49
38
28
17
14
B
Overall Rank
# of LaGs Identified
No Digestion
Papain
IdeS
C
V$_H$H sequence coverage (%)
CDR3 sequence coverage (%)
Number of CDR3 groups
Trypsin
Chymo
Trypsin + Chymo
D
Gel slices of V$_H$H band:
19 kDa
Unique PSMs from gel slices:
Slice 1 1430
Slice 2 2727
Slice 3 6351
Slice 4 5852
Slice 5 4250
Slice 1-5 12027
Non-Sliced 7795
Relative intensity of CDR peptides from gel slices:
Nb1 CDR1
Nb1 CDR2
Nb1 CDR3
Nb2 CDR1
Nb2 CDR2
Nb2 CDR3
Slice 1
Slice 2
Slice 3
Slice 4
Slice 5

Figure S2

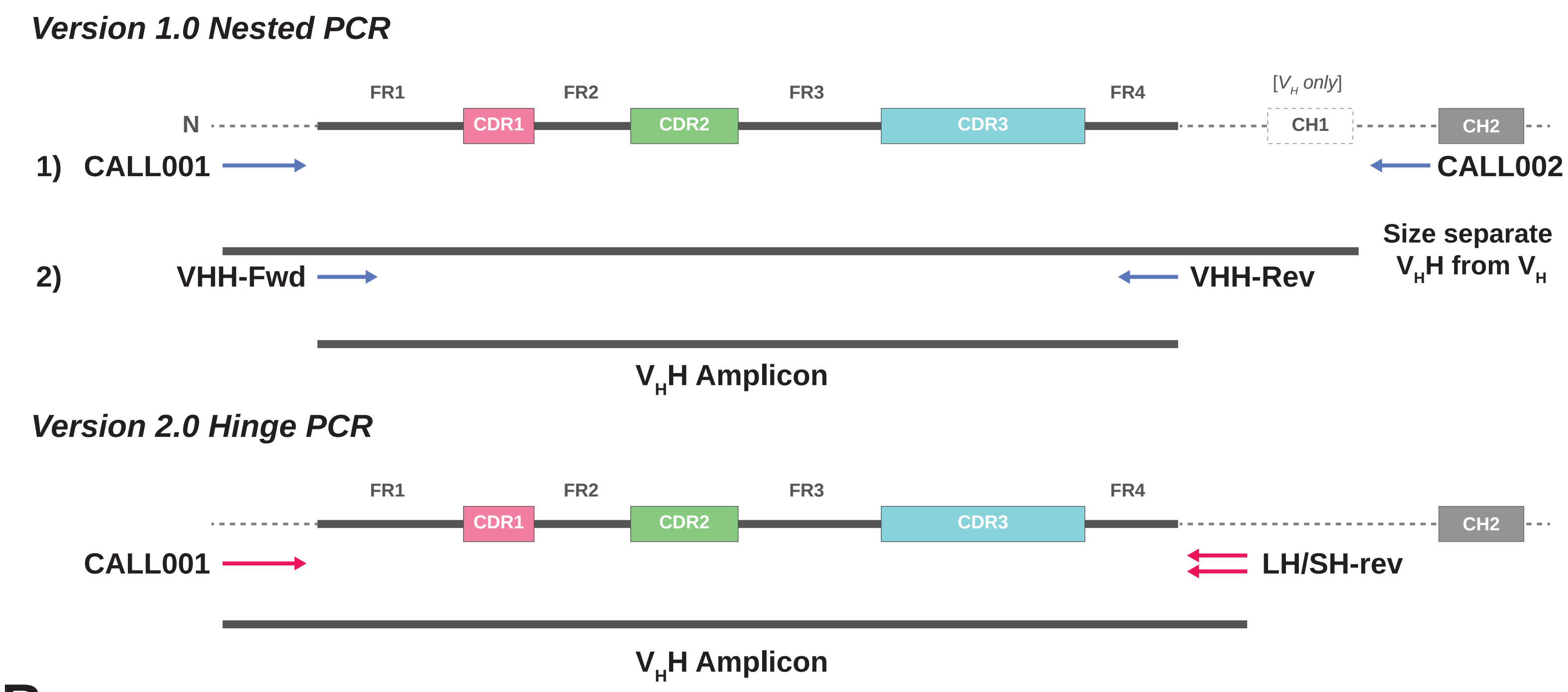

B

Llama V_HH

N-term V_HH (FR1)	%	C-term V_HH (FR4)	%
caggtgcagctggtggag	8.1%	acccaggtcaccgtctcc	62.5%
caggtgcagctggtagag	7.6%	accctggtcaccgtctcc	6.6%
caggtgcagttggtggag	4.4%	acccaggtcaccgtctct	2.9%
gaggtgcagctcgtggag	4.0%	actcaggtcaccgtctcc	2.6%
caggtgcaggtggtggaa	3.0%	atcctggtcaccatctcc	1.9%
caggtgcagctcgtggag	2.5%	acccaagtcaccgtctcc	1.7%
caggtgcagctggcagag	2.4%	acccaggtcaccgtcgcc	1.6%
caggtgcacctggtagag	2.3%	acgcaggtcaccgtctcc	1.2%
caggtgcaactggtggag	2.0%	atccaggtcaccgtctcc	1.1%
caggtgcagttggtagag	1.6%	acccaggtcactgtctcc	1.1%
caggtgcatttggtggag	1.4%	acccaggtcaccgtctcg	1.1%
caggtacagctcgtggag	1.4%	acccgggtcaccgtttcc	1.0%
caggtgcaactggtagag	1.3%	acccaggttaccgtctcc	1.0%
caggtgcagctggtagaa	0.7%	acccaggtcaccgtttcc	0.9%
gaggagccactcgtggag	0.7%	attcaggtcaccgtctcc	0.9%
gcggtgcagctcgtggag	0.7%	acccgggtcaccgtctcc	0.7%
caggtgcaggtcgtggag	0.6%	acccaggtcaccgtctca	0.7%
gaggtgcagctggtggag	0.6%	accctggtcaccgtttcc	0.7%
caggtgcaggtggtggag	0.6%	accctggtcactgtctcc	0.6%
caggtgcacctggtggag	0.6%	acccaggtgaccgtctcc	0.6%
**Top 20 Total:**	47%	**Top 20 Total:**	92%

Alpaca V_HH

N-term V_HH (FR1)	%	C-term V_HH (FR4)	%
caggtgcagctggtggag	12.1%	acccaggtcaccgtctcc	63.7%
cagttgcagctcgtggag	5.8%	accctggtcaccgtctcc	4.3%
caggtgcagctcgtggag	4.5%	acccaggtcaccgtctct	3.2%
gaggtgcagctcgtggag	4.2%	actcaggtcaccgtctcc	3.2%
caggtgcagttggtggag	2.0%	acccaggtcactgtctcc	1.7%
caggtgcaactggtggag	1.6%	accgaggtcaccgtcacc	1.7%
cagttgcaactcgtggag	1.5%	atccaggtcaccgtctcc	1.6%
caggtgcaggtgatggaa	1.3%	acccaggtcaccgtttcc	1.5%
gaaatacagttcgtggag	1.2%	acccaagtcaccgtctcc	1.4%
gaggtgcagctggtggag	1.0%	acccaggtcaccgtcgcc	1.3%
cagttgcagttcgtggag	0.8%	acccgggtcaccgtctcc	1.3%
cagttgcggctcgtggag	0.6%	acccaggtcatcgtctcc	1.2%
caggtgcacctggtggag	0.6%	acccaggtcaccgtctcg	1.1%
caggtgcagctggcggag	0.6%	acccaggttaccgtctcc	0.9%
gcgttgcagctcgtggag	0.6%	acctcggtcaccgtttcc	0.9%
gaggtgcggatcgtggag	0.5%	acgcaggtcaccgtctcc	0.7%
caggtgccgctggtggag	0.5%	accctggtcaccgtctct	0.6%
cagttgcaggtcgtggag	0.5%	acccaggtgaccgtctcc	0.6%
caggtgcaactcgtggag	0.5%	acccaggtcaccgtgtcc	0.5%
caggagcaagtgaaggag	0.5%	acccaggtcaccgtctca	0.5%
**Top 20 Total:**	41%	**Top 20 Total:**	92%

C

CALL001 (N-term Leader):	%	SH-rev (Short Hinge):	%	LH-rev (Long Hinge):	%
gtcctggctgctctttacaagg	25.1%	gcaccacagcgaagaccccag	67.5%	cccaagacaccaaaaccacaaccac	86.9%
gtcctggctgctctactacaagg	23.3%	* gcacccagcgaacccccac	8.5%	cccaagacaccaaaaccacacccac	1.4%
gtcctggctgctcttctacaagg	21.4%	* gccctacagggaggaccccag	4.7%	cccaagccaccaaaaccacaaccac	1.2%
gtcctgggtgctcttctacaagg	5.3%	gcaccacagcgaagcccccag	3.2%	cccaagacaccaaaaccacaacccc	0.8%
gtcctggctgctcttgtacaagg	3.9%	* gcaccaccgtggagaccgcag	2.8%	cccaagacaccaaacccacaaccac	0.7%
ttcctgggtgctcttctacaagg	3.2%	* tcaccacagtgacgacccaag	1.3%	cccaagacaccaaaaccaccaccac	0.7%
gtactggctgctctactacaagg	1.7%	gcaccacagcgaagaccccccg	1.0%	cccaacacaccaaaaccacaaccac	0.5%
gtgctggcggcccttctgctagg	1.2%	* gccccacagcgaagaccccag	0.9%	cccaagacaccaaaaccaaaaccac	0.5%
gtcctggctgctctcctacaagg	0.9%	* aaaccacagccaagaccacag	0.7%	cccaagacaccaaaacccccaaccac	0.5%
gtcctggctgctctactacaggg	0.8%	* gcaccacagtgaattccctag	0.6%	cccaagacaccaacaccacaaccac	0.5%
**Top 10 Total:**	87%	**Top 10 Total:**	91%	**Top 10 Total:**	94%

*Adjacent to non-FR4/hinge sequence

Figure S3

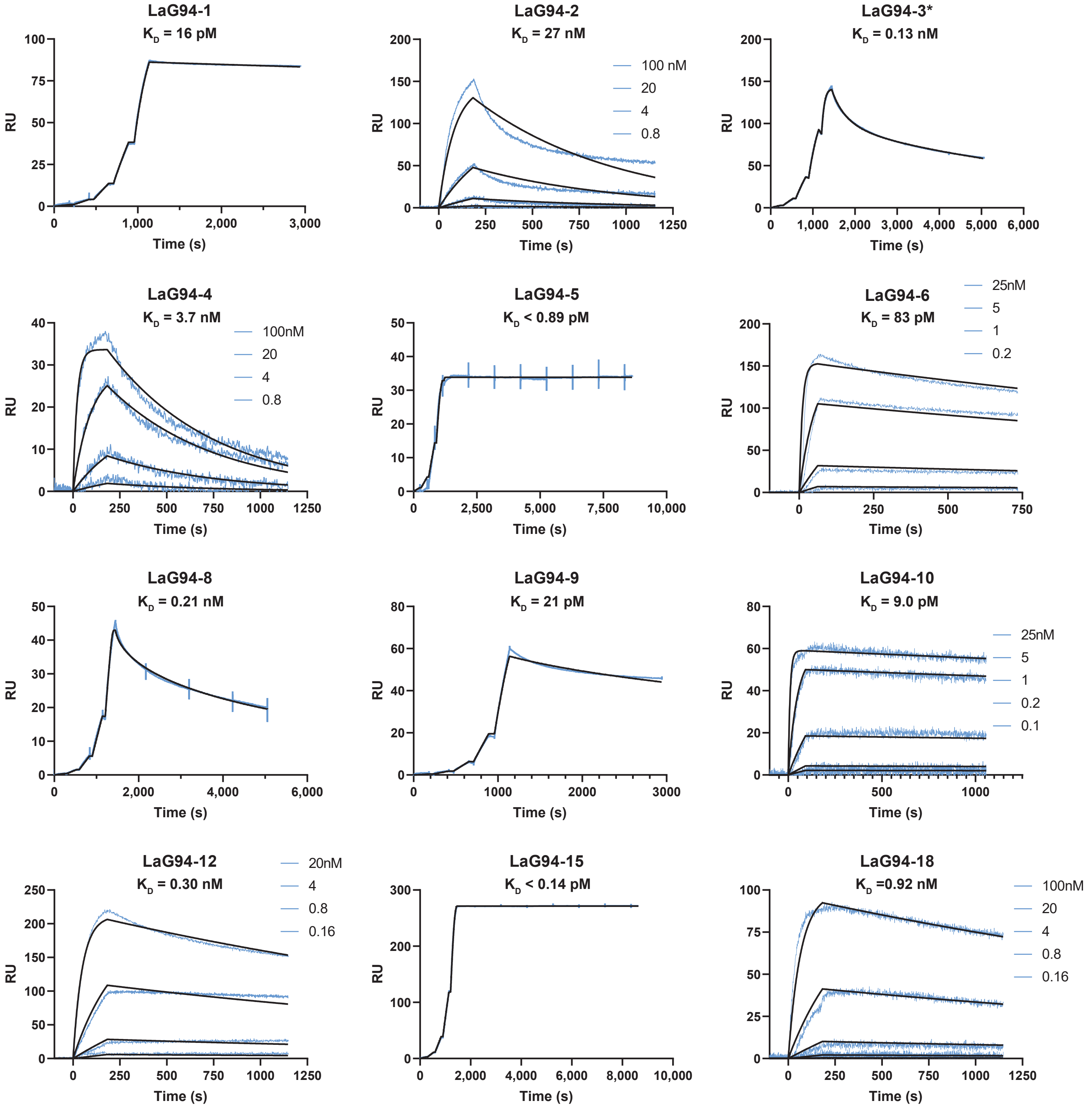

LaG94-1
K_D = 16 pM
LaG94-2
K_D = 27 nM
100 nM
20
4
0.8
LaG94-3*
K_D = 0.13 nM
LaG94-4
K_D = 3.7 nM
100nM
20
4
0.8
LaG94-5
K_D < 0.89 pM
LaG94-6
K_D = 83 pM
25nM
5
1
0.2
LaG94-8
K_D = 0.21 nM
LaG94-9
K_D = 21 pM
LaG94-10
K_D = 9.0 pM
25nM
5
1
0.2
0.1
LaG94-12
K_D = 0.30 nM
20nM
4
0.8
0.16
LaG94-15
K_D < 0.14 pM
LaG94-18
K_D =0.92 nM
100nM
20
4
0.8
0.16
RU
Time (s)

Figure S4

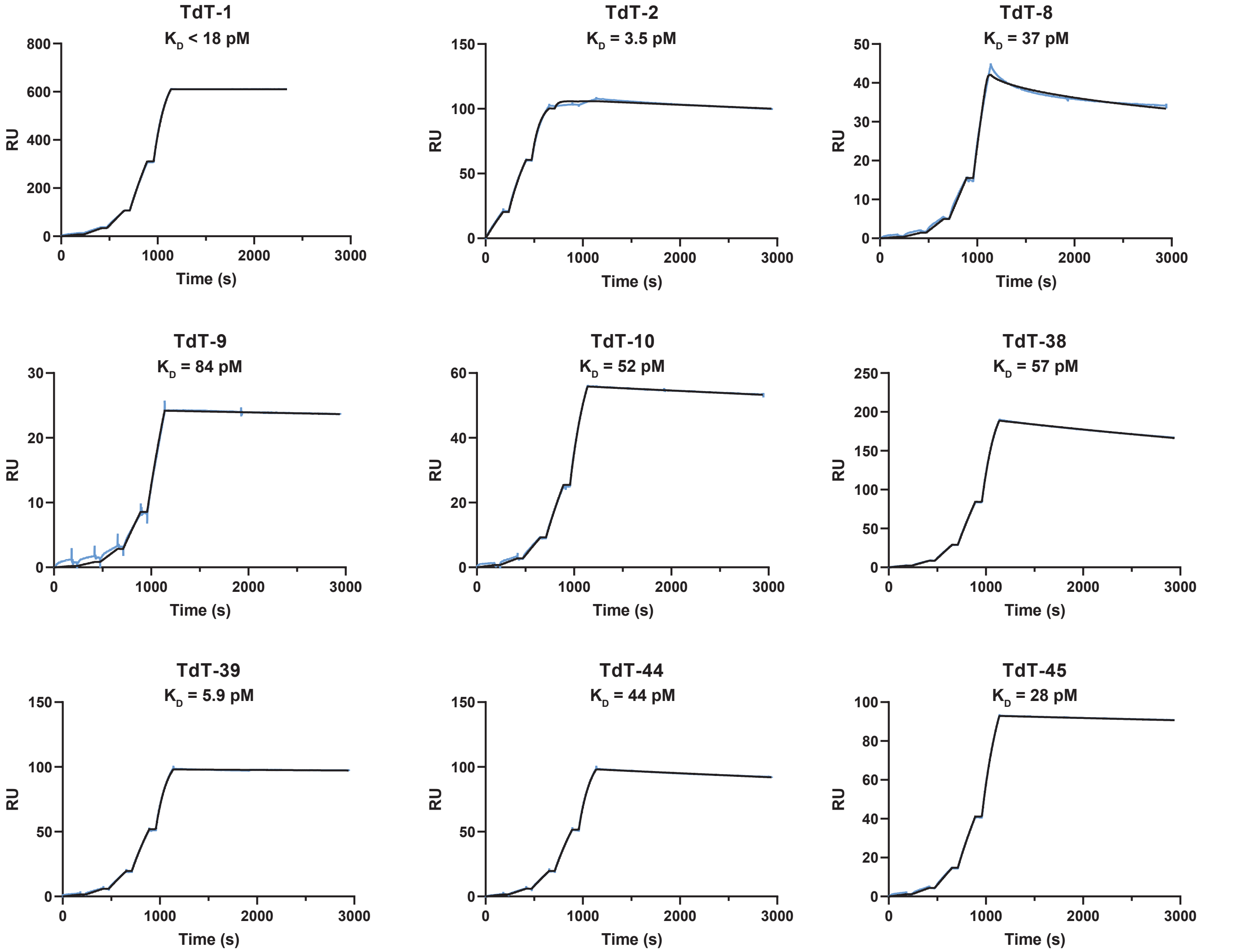

TdT-1
K_D < 18 pM
TdT-2
K_D = 3.5 pM
TdT-8
K_D = 37 pM
TdT-9
K_D = 84 pM
TdT-10
K_D = 52 pM
TdT-38
K_D = 57 pM
TdT-39
K_D = 5.9 pM
TdT-44
K_D = 44 pM
TdT-45
K_D = 28 pM
RU
Time (s)

Figure S5

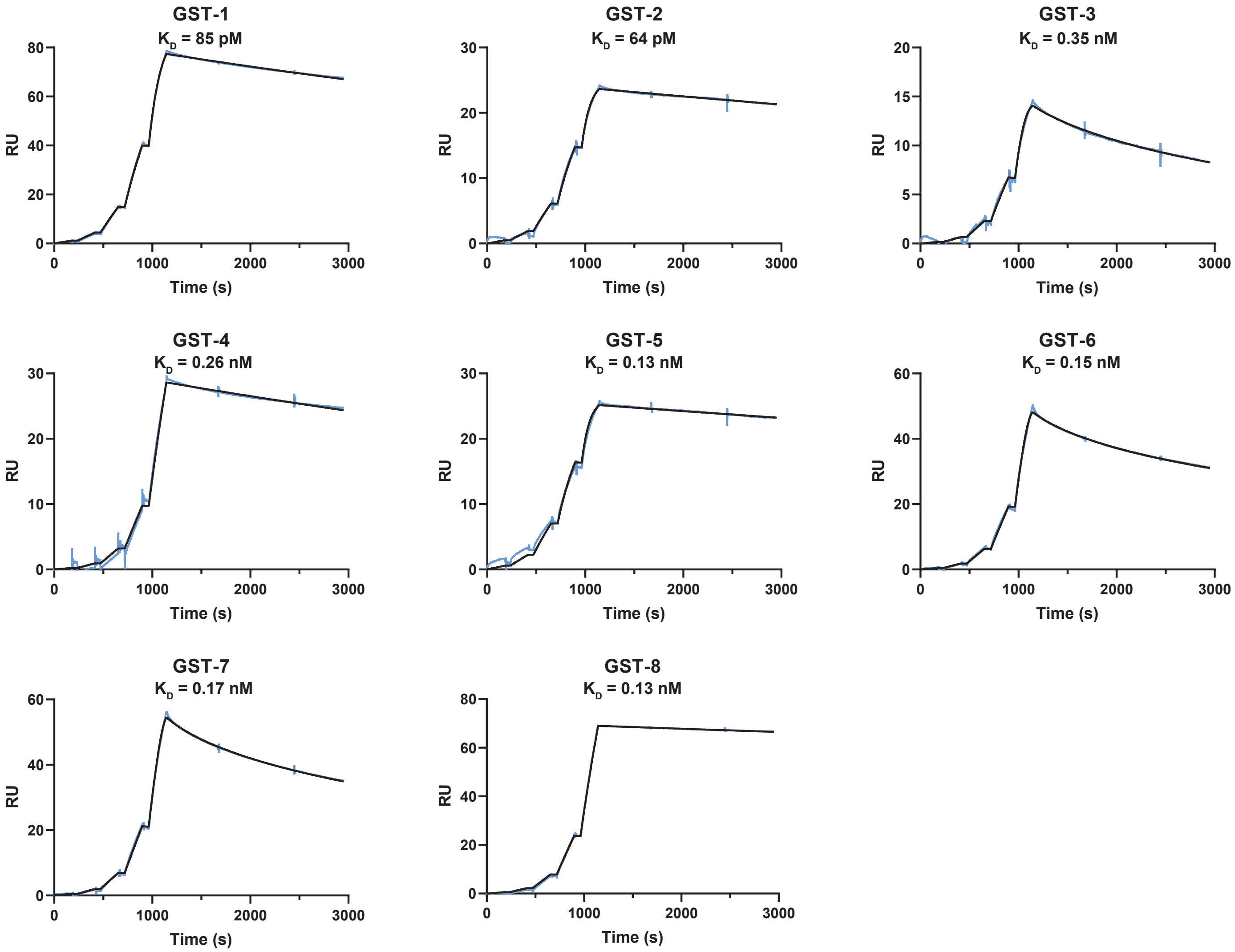
GST-1
K_D = 85 pM
GST-2
K_D = 64 pM
GST-3
K_D = 0.35 nM
GST-4
K_D = 0.26 nM
GST-5
K_D = 0.13 nM
GST-6
K_D = 0.15 nM
GST-7
K_D = 0.17 nM
GST-8
K_D = 0.13 nM
RU
Time (s)

Figure S6

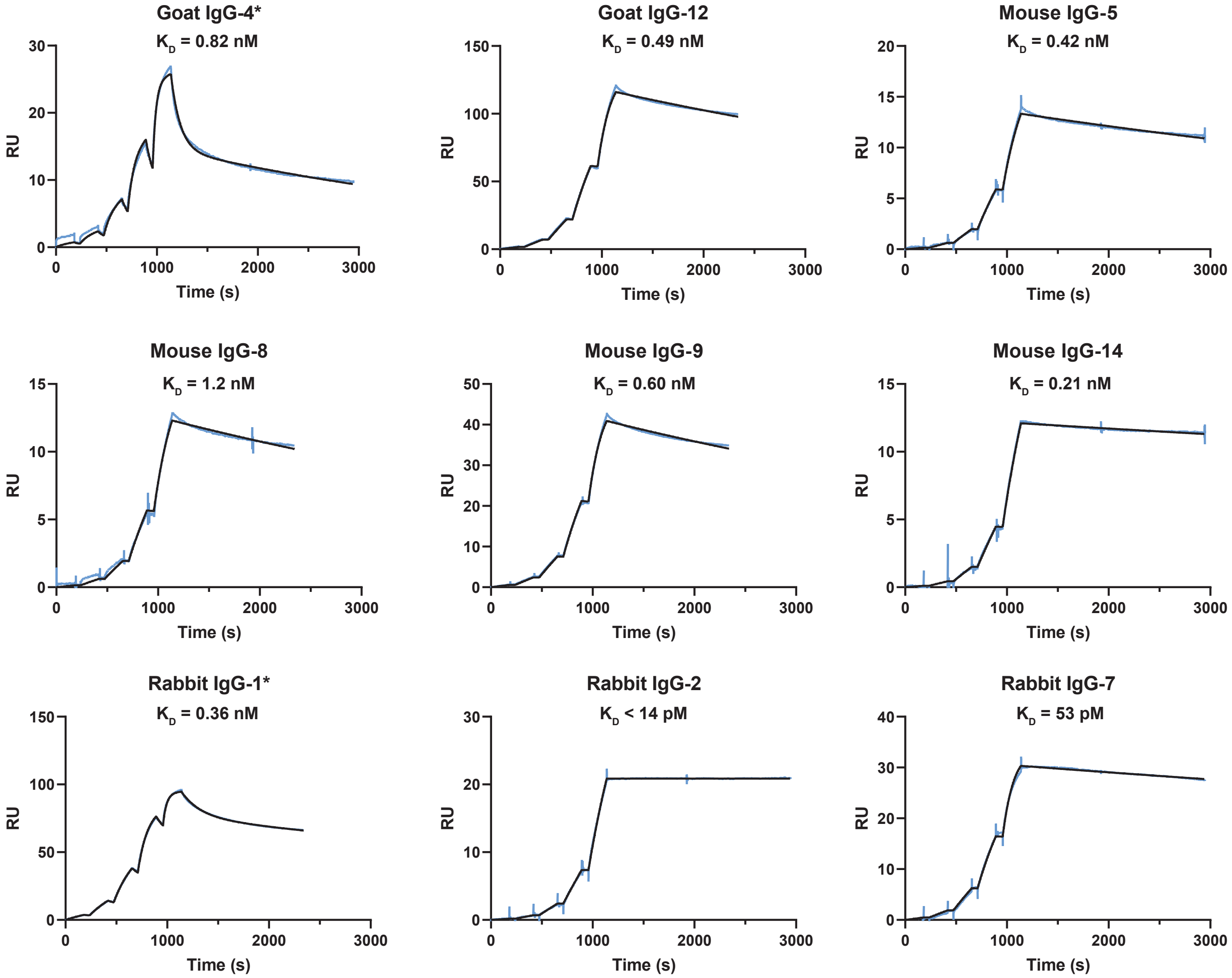
Goat IgG-4*
K_D = 0.82 nM
Goat IgG-12
K_D = 0.49 nM
Mouse IgG-5
K_D = 0.42 nM
Mouse IgG-8
K_D = 1.2 nM
Mouse IgG-9
K_D = 0.60 nM
Mouse IgG-14
K_D = 0.21 nM
Rabbit IgG-1*
K_D = 0.36 nM
Rabbit IgG-2
K_D < 14 pM
Rabbit IgG-7
K_D = 53 pM
RU
Time (s)

Figure S7

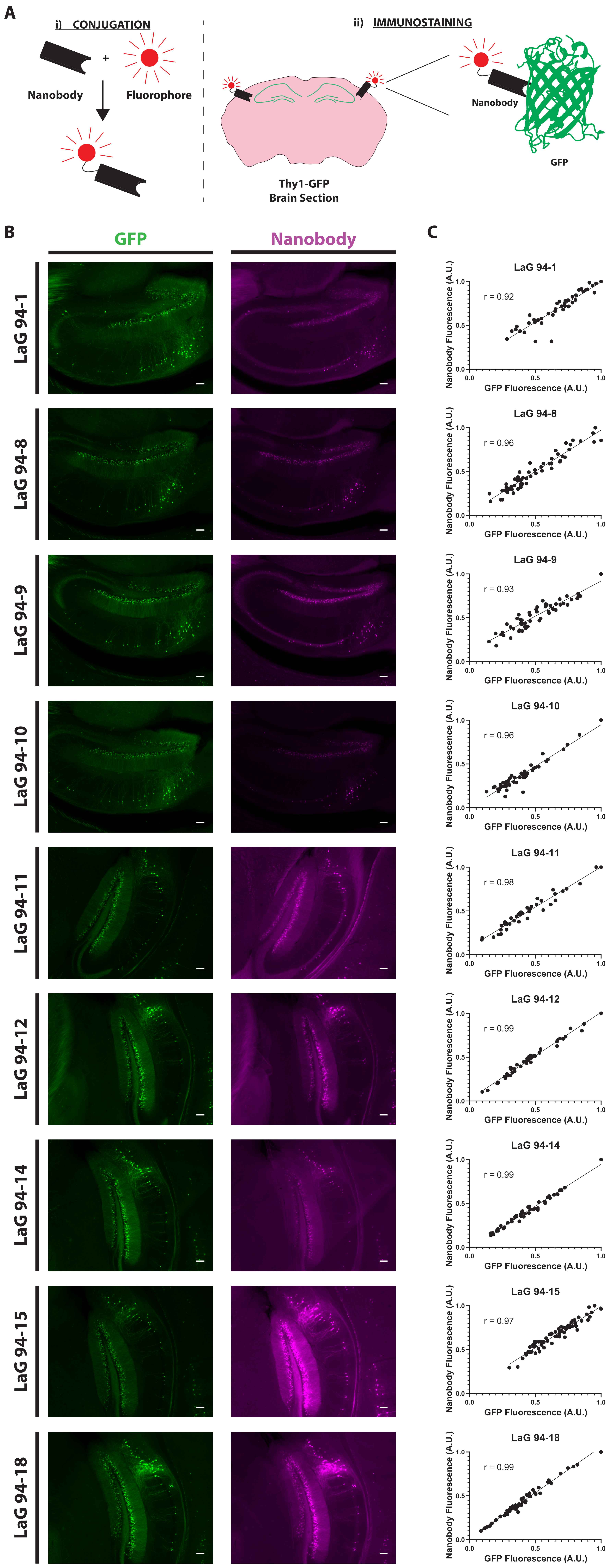
A
i) CONJUGATION
Nanobody
Fluorophore
ii) IMMUNOSTAINING
Thy1-GFP
Brain Section
Nanobody
GFP
B
GFP
Nanobody
LaG 94-1
LaG 94-8
LaG 94-9
LaG 94-10
LaG 94-11
LaG 94-12
LaG 94-14
LaG 94-15
LaG 94-18
C
LaG 94-1
r = 0.92
LaG 94-8
r = 0.96
LaG 94-9
r = 0.93
LaG 94-10
r = 0.96
LaG 94-11
r = 0.98
LaG 94-12
r = 0.99
LaG 94-14
r = 0.99
LaG 94-15
r = 0.97
LaG 94-18
r = 0.99
Nanobody Fluorescence (A.U.)
GFP Fluorescence (A.U.)

Figure S8

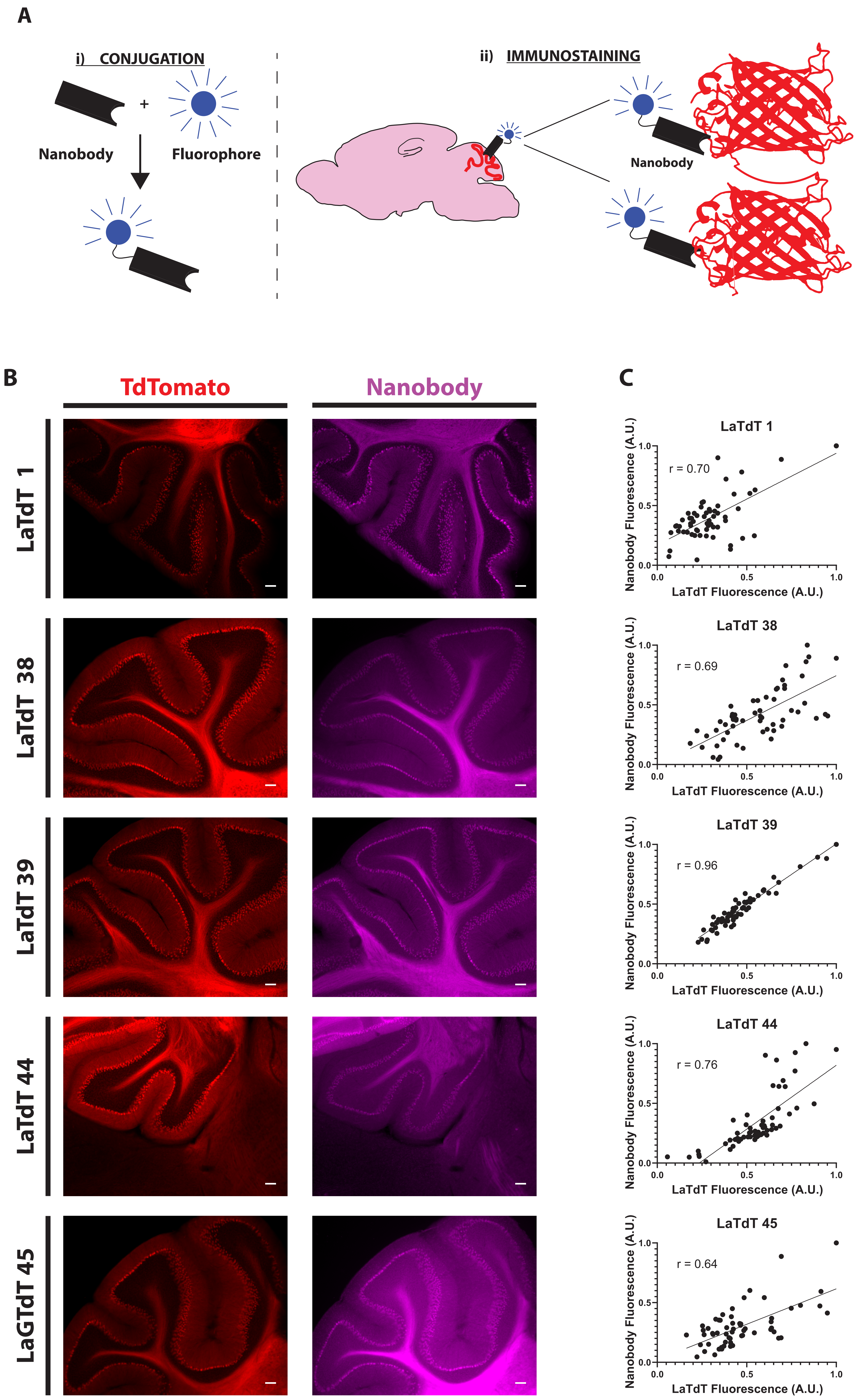
A
i) CONJUGATION
+
Nanobody
Fluorophore
ii) IMMUNOSTAINING
Nanobody
B
TdTomato
Nanobody
LaTdT 1
LaTdT 38
LaTdT 39
LaTdT 44
LaGTdT 45
C
LaTdT 1
r = 0.70
LaTdT 38
r = 0.69
LaTdT 39
r = 0.96
LaTdT 44
r = 0.76
LaTdT 45
r = 0.64
Nanobody Fluorescence (A.U.)
LaTdT Fluorescence (A.U.)
0.0
0.5
1.0

Figure S9

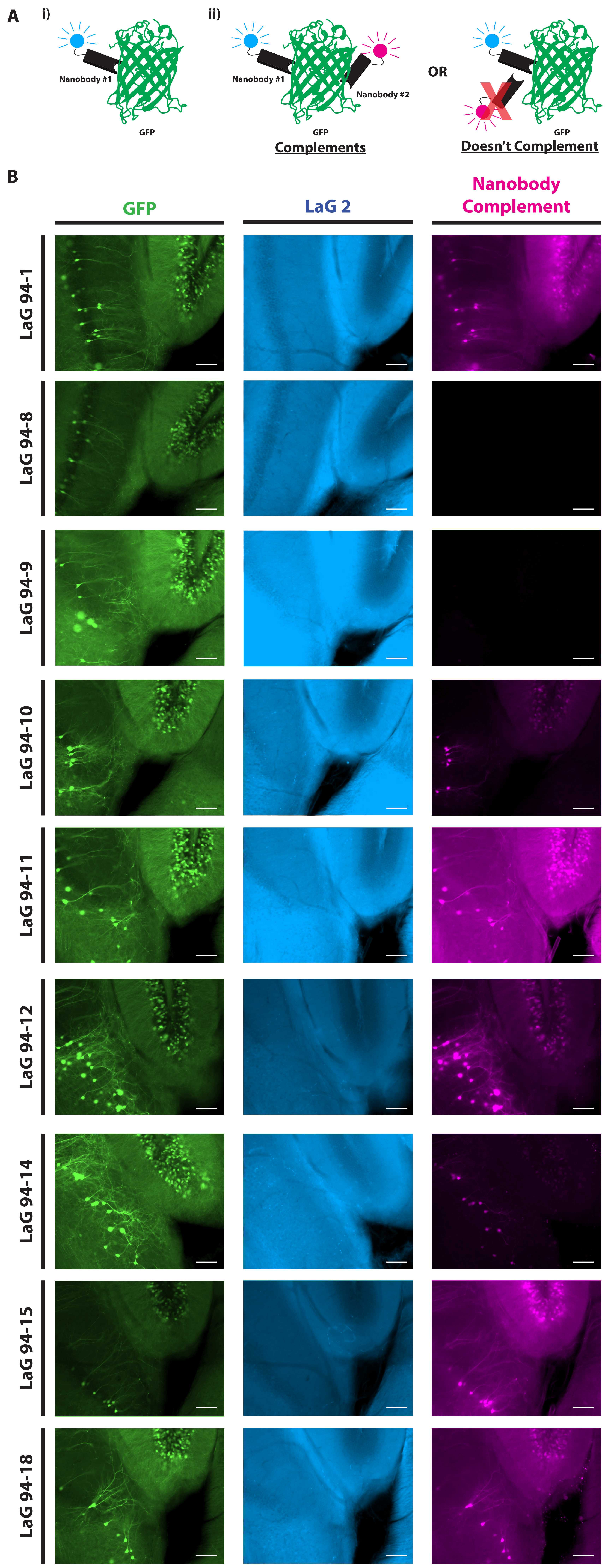
A
i)
Nanobody #1
GFP
ii)
Nanobody #1
Nanobody #2
GFP
Complements
OR
GFP
Doesn't Complement
B
GFP
LaG 2
Nanobody Complement
LaG 94-1
LaG 94-8
LaG 94-9
LaG 94-10
LaG 94-11
LaG 94-12
LaG 94-14
LaG 94-15
LaG 94-18

Figure S10

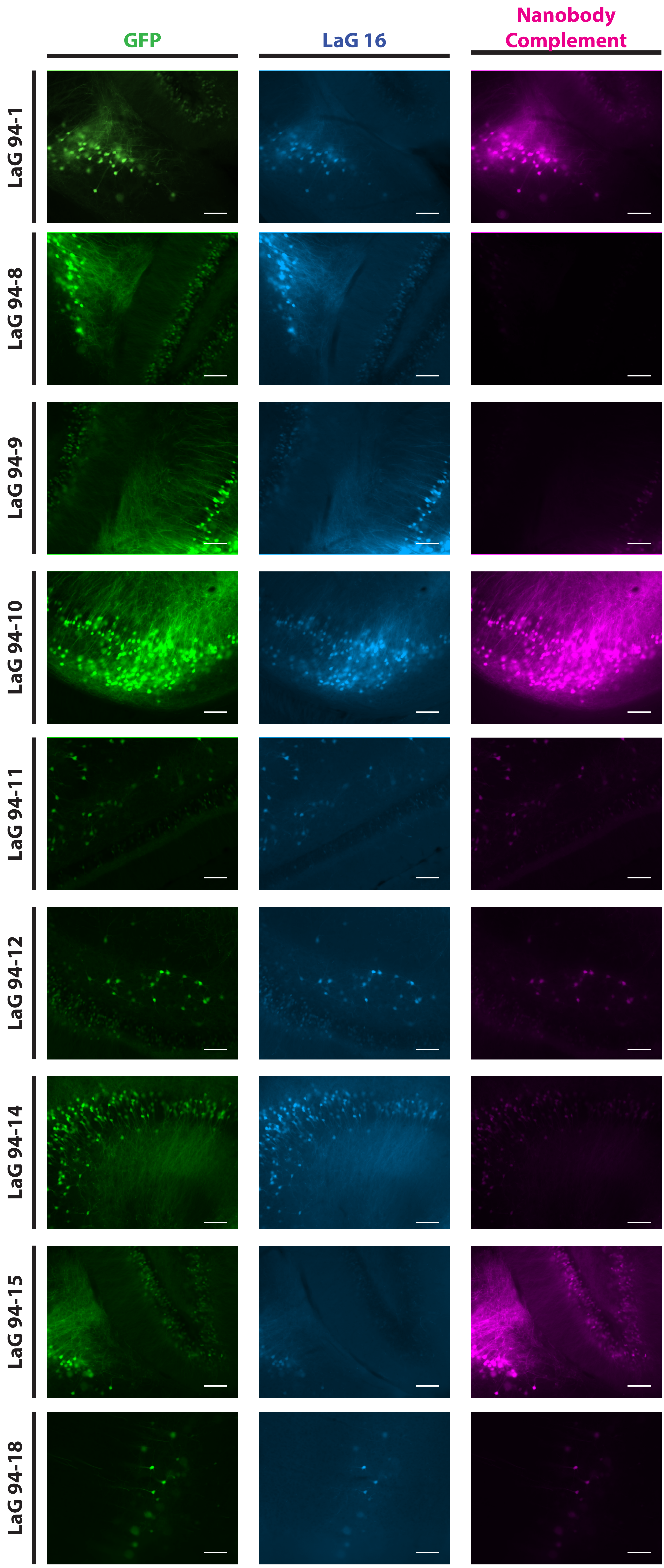
GFP
LaG 16
Nanobody Complement
LaG 94-1
LaG 94-8
LaG 94-9
LaG 94-10
LaG 94-11
LaG 94-12
LaG 94-14
LaG 94-15
LaG 94-18

Figure S11

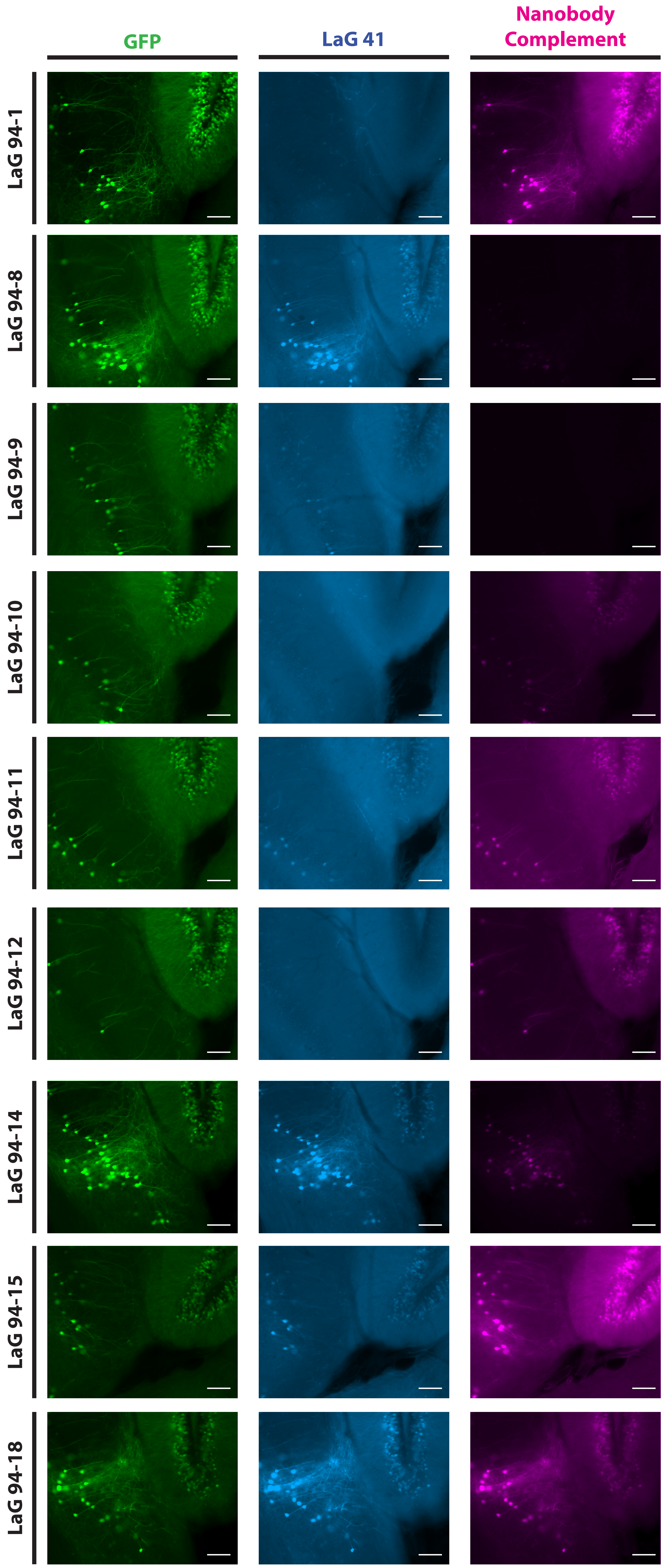
GFP
LaG 41
Nanobody Complement
LaG 94-1
LaG 94-8
LaG 94-9
LaG 94-10
LaG 94-11
LaG 94-12
LaG 94-14
LaG 94-15
LaG 94-18

Figure S12

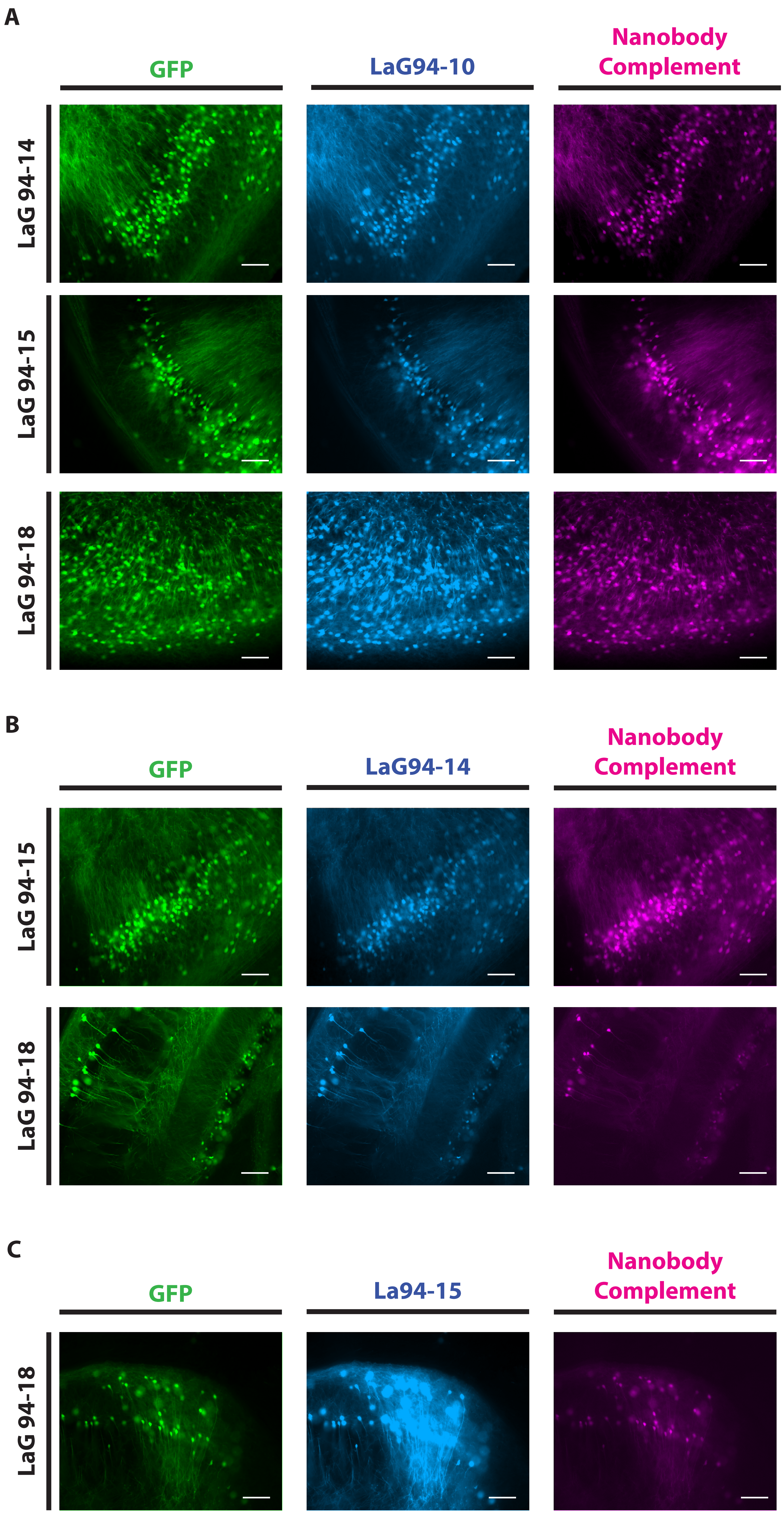
A
GFP
LaG94-10
Nanobody Complement
LaG 94-14
LaG 94-15
LaG 94-18
B
GFP
LaG94-14
Nanobody Complement
LaG 94-15
LaG 94-18
C
GFP
La94-15
Nanobody Complement
LaG 94-18

Figure S13

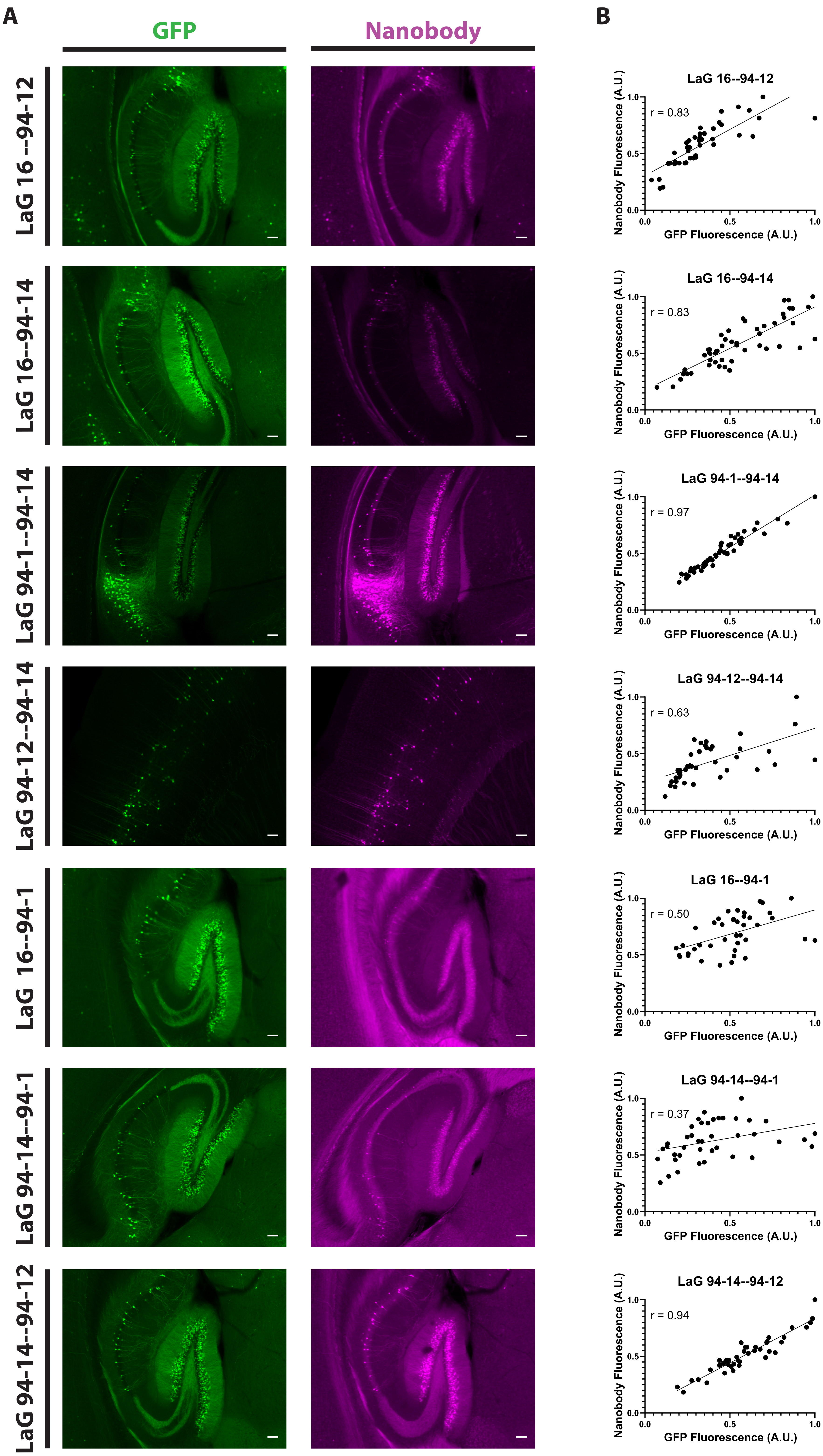

A
GFP
Nanobody
LaG 16--94-12
LaG 16--94-14
LaG 94-1--94-14
LaG 94-12--94-14
LaG 16--94-1
LaG 94-14--94-1
LaG 94-14--94-12
B
LaG 16--94-12
r = 0.83
LaG 16--94-14
r = 0.83
LaG 94-1--94-14
r = 0.97
LaG 94-12--94-14
r = 0.63
LaG 16--94-1
r = 0.50
LaG 94-14--94-1
r = 0.37
LaG 94-14--94-12
r = 0.94
Nanobody Fluorescence (A.U.)
GFP Fluorescence (A.U.)

Figure S14

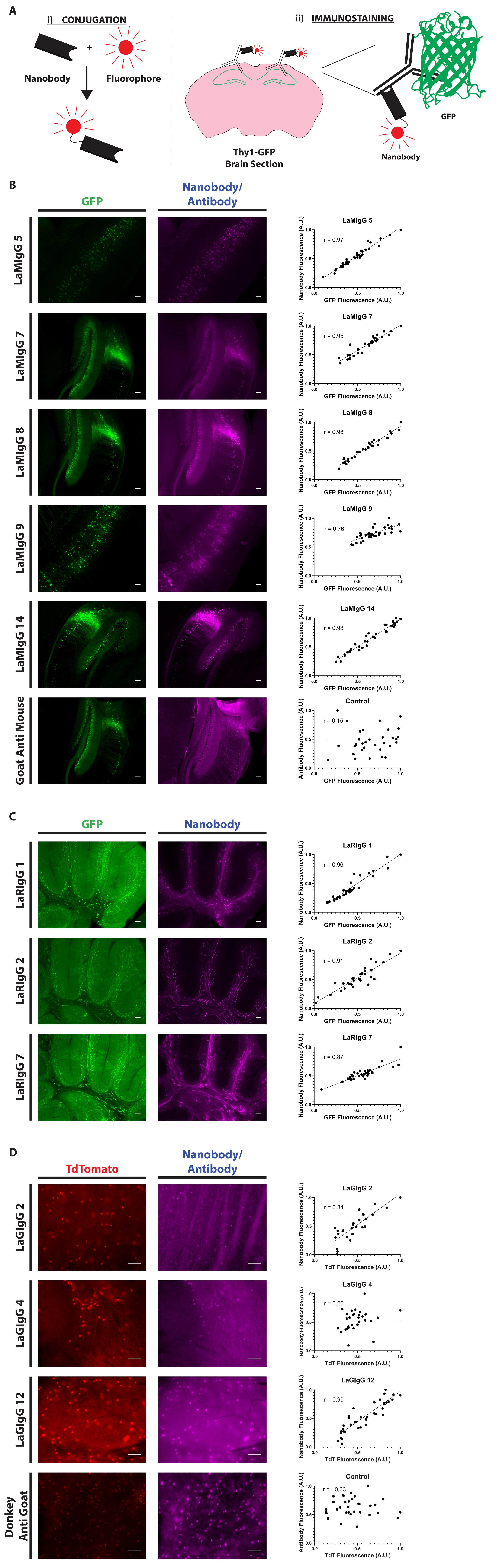

A
i) CONJUGATION
Nanobody
Fluorophore
ii) IMMUNOSTAINING
Thy1-GFP
Brain Section
GFP
Nanobody
B
GFP
Nanobody/
Antibody
LaMIgG 5
LaMIgG 7
LaMIgG 8
LaMIgG 9
LaMIgG 14
Goat Anti Mouse
r = 0.97
r = 0.95
r = 0.98
r = 0.76
r = 0.98
Control
r = 0.15
Nanobody Fluorescence (A.U.)
Antibody Fluorescence (A.U.)
GFP Fluorescence (A.U.)
C
GFP
Nanobody
LaRIgG 1
LaRIgG 2
LaRIgG 7
r = 0.96
r = 0.91
r = 0.87
D
TdTomato
Nanobody/
Antibody
LaGIgG 2
LaGIgG 4
LaGIgG 12
Donkey
Anti Goat
r = 0.84
r = 0.25
r = 0.90
r = - 0.03
TdT Fluorescence (A.U.)

Figure S15

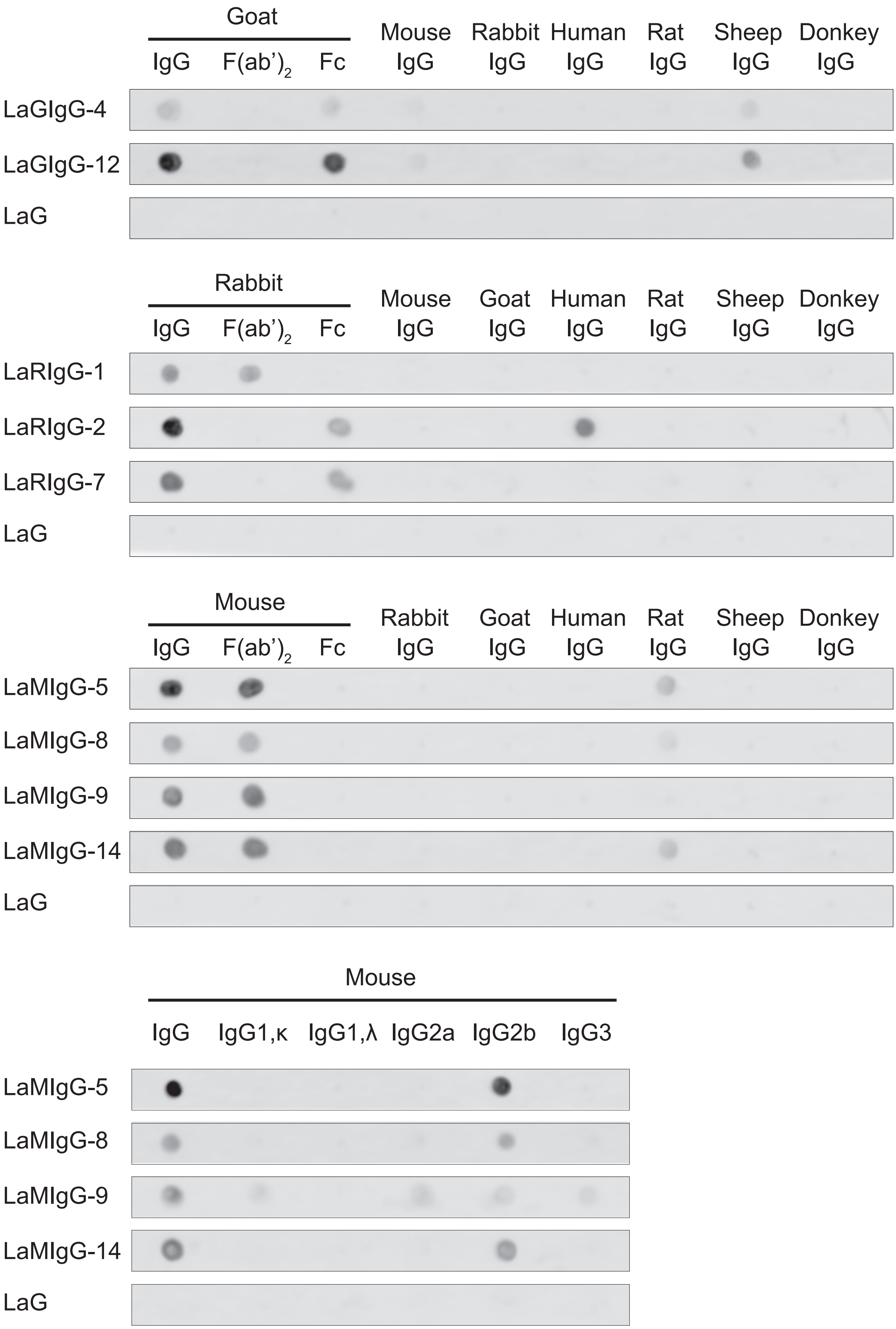
Goat
IgG
F(ab')2
Fc
Mouse IgG
Rabbit IgG
Human IgG
Rat IgG
Sheep IgG
Donkey IgG
LaGIgG-4
LaGIgG-12
LaG
Rabbit
IgG
F(ab')2
Fc
Mouse IgG
Goat IgG
Human IgG
Rat IgG
Sheep IgG
Donkey IgG
LaRIgG-1
LaRIgG-2
LaRIgG-7
LaG
Mouse
IgG
F(ab')2
Fc
Rabbit IgG
Goat IgG
Human IgG
Rat IgG
Sheep IgG
Donkey IgG
LaMIgG-5
LaMIgG-8
LaMIgG-9
LaMIgG-14
LaG
Mouse
IgG
IgG1,κ
IgG1,λ
IgG2a
IgG2b
IgG3
LaMIgG-5
LaMIgG-8
LaMIgG-9
LaMIgG-14
LaG

Table S1. Nanobody amino acid sequences.

Nanobody ID	Amino acid sequence
LaG94-1	MAQVQLVESGGGLVQTGDSLRLSCRASGLTLSVYSTGWFRQAPGKEHENVAWISRSGDETYYEDSVKGRFTISRDNAKNTVYLQMNDLKPEDMARYYCAASNRGRTTAWNLYIYRGQGTQVTVS
LaG94-2	MADVQLVESGGALVQAGGSLRLSCEFSGGTFNLYGIGWFRQAPEQDHEFVAGVSRYGSTYESVSVKGRFTSSRDNAKNSAYLEMNTLKPEDTGVYTCAATVREQVPTNFGSYIYRGQGTQVTVS
LaG94-3	MAQVQLVESGGGLVQAGGSLRLSCTASGGTVSSSGMGWFRQSSGNDRDFVAFITPTGPTTWYEESVNSRFTVSRDNAKNTIYLQMKSLRPEDTAIYYCAAGNIGRDSRTYPYWGQGTQVTVS
LaG94-4	MADVQLVESGGGLVQAGGSLRLSCAASGDTSSIHFWEWYRQAPGKQRELVATANNGDFTHLQDSVKGRFTISKDNAKSTVYLQMNDLKPEDTAVYLCYARSYSNSNFWGQGTQVTVS
LaG94-5	MAQVQLAESGGGLVQAGGSLRLSCAASGRTFAMGWFRRAPGKEREFVASMSRSGLDTHYADSVKGRFTISRDNALNTVYLQTDSLKPEDTGDYFCAVGLRYWSLSGDYIRREKYDVWGQGTQVTVS
LaG94-6	MAQVQLVESGGGAVQAGGSLRLSCAASGLTFSLYTMGWFRQAPEKEPEFVAYISKSGDTTTYADSVKGRFTIGRDNAKSTVYLQMNSLKSEDTAVYYCAASIKREITPNSIYRGQGTQVTVS
LaG94-8	MAQVQLVESGGGLVQAGGSLRLSCAASGRTFMNYAMGWFRQAPGKGREFVASISWTGETSTYADFVKGRFTISRDNAKNTVYLQMDSLKPEDTAVYRCAAKRATDFDDTVREAAEADYWGQGTQVTVS
LaG94-9	MAQVQLVESGGGLVQPGGSLRLSCAASGGTFDTHTMGWFRQAPGKQREFVSAIRLHGVGTYYADSVKGRFTISRENAKNTVHLQMNNLKPEDTAVYYCAADRNWSRATEKEEYDYWGQGTQVTVS
LaG94-10	MAQVQLVESGGGLVQAGGSLRLSCAASGLTLRVYDVTWFRQAPGKEREFVGTVNRNGVVTDYADSVKGRFTISRDNPKNTIYLQMNSLKPEDTAVYYCAAKTRPKLSTLWDEYIYRGQGTQVTVS
LaG94-12	MAQVQLVESGGRLVQAGGSLRLSCAVSGGTFSLYSLGWFRQAPGKEREFVAAFSREDGTTSFADSVKGRFTMFRDGTKNTVYLQMNSLKQEDTAVYSCAATVRAQPSYSWSSYVYRGLGTQVTVS
LaG94-15	MAQVQLVESGGGAVQSGGSLSLSCAASGGIRSISAMTWFRQAPGREREFVAAVTRTLGTTYYTDSVKGRFTISRDSVKGTLYLRMNNMRPEDAAVYYCAARTRGFSTKVALETTDGMDYWGKGTLVTVS
LaG94-18	MAQVQLVESGGGLVQAGGSLRLSCVASGRTFSTSAMAWWRQAPGKEREFVAAISWNVGTTYYPDSVKGRFTISRDKAQNAVYLQMNSLKPEDTAVYYCAARVTGYFGDQVRRADDYRYWGQGTQVTVS
LaTdT-1	MADVQLVESGGGLVPAGGSLRLSCAASTSDLFSRYTMGWFRQAPGKEREFVSAITGMSSMTKYADSVKGRFTVSRDNRKATMYLQMDSLKPEDTAVYYCAAHTTTAAATPQSMGLMLKYDHWGQGTEVTVS
LaTdT-2	MADVQLVESGGGLVQAGDSLRLSCAFSGSAFDTYAMGWFRQAPGKEREFVSAITWSGGNTYYADSAKGRFTISRDDAKNTVYLQMNSLKPEDAATYYCAANRYGSSWSPLTKMGQYEHWGQGTQVTVS
LaTdT-8	MAQVQLVESGGGLVQAGDSLRLSCADAGLPYAFNHYGMAWFRQAPGKEREFVAGTSGGMTYYGDSVPDRFSFSRDNDKKTVHLHINSLKPEDTAVYYCAARLGGRYEDASDYDVWGQGTQVTVS

LaTdT-9	MAQVQLVESGGGLVQPGDSLMLSCVISGHTFTNFRMGWFRQAPGKEREFVAGITWLFGRTDYADSVQGRFTISRDKGKDTMYLQMDSLKPEDTGVYTCAVQSGVLAYPMREGFYDIWGQGTHVTVS
LaTdT-10	MADVQLVESGGRLVQDGGSLRLSCEVSGGTFDTFAMGWFRQAPGKEREFVSAITWSGGNTYYGDSVKGRFTISRNNVKRTVYLQMISLKPEDTGVYYCAADPYGSSWSPLQKEGQYDYWGQGTQVTVS
LaTdT-38	MAQVQLVESGGGLVQAGGSLRLSCAASGRSFSGNNMGWFRQVSGTEREFVAAISWSGLITEYSGAVKGRFTISRDNAKNTLYLQMNSLKPEDTAVYSCAADGRDVQLPGRSEYDYNYWGQGTQVTVS
LaTdT-39	MAQVQLVESGGGLVQAGESLQLSCAASGGTLRDAIVGWFRQARGKEREFLASITWSGLSTNYSDSVKGRFVISRANPQNTAYLQMNSLKPEDTAIYYCAAKIGAFGLADEFRTVPQYDSWGQGTQVTVS
LaTdT-44	MAQQVQLVESGGRLVQDGGSLRLSCEVSGGTFDTFAMGWFRQAPGKEREFVSAITWSGGNTYYGDSVKGRFTISRNNVKRTVYLQMISLKPEDTGVYYCAADPYGSSWSPLQKEGQYDYWGQGTQVTVS
LaTdT-45	MTQVQLVESGGGLVQAGDSLRLSCAFSGSAFDTYAMGWFRQAPGKEREFVSAITWSGGNTYYADSAKGRFTISRDDAKNTVYLQMNSLKPEDAATYYCAANRYGSSWSPLTKMGQYEHWGQGTQVTVS
GST-1	QVQLVESGGGLVQAGGSLRLSCVCVGRAFGVYAMGWFRQSPGKEREFVAAITWNGGSIDYADSVKGRFAISRDNPPNTVYLQMNSLKPEDTAVYYCAKYRNNYYNEAPNSWGQGTQVTVS
GST-2	QVQLVESGGGLVQAGGSLRLSCVVVGRAFGAYAMGWFRQSPGKEREFVAAITWNGGSITYADSVKGRFAISRDNPPNTVYLEMNSLKPEDTAVYYCAKYTNNYYNEAPKSWGQGTQVTVS
GST-3	QVQLVESGGGLVQPGGSLRLSCADSGRIFSDYTMAWFRQPPGKEREFVASINRSGLGTYYADSVKGRFTVTRDNAKNMVYLQMNSLTVEDTAVYYCGARWGGSYIDPREGMYDFRGQGTQVTVS
GST-4	QVQLVESGGGLVQAGGSLRLSCVCVGRAFGVYAMGWFRQSPGKEREFVAAITWNGGSIDYADSVKGRFAISRDNPPNTVYLQMNSLKPEDTAVYYCAKYRNNYYNEAPNSWGQGTQVTVS
GST-5	QVQLVESGGGLVQAGGSLRLSCVVTGRAFGAYAMGWFRQSPGKEREFVAAITWNGGTTNYADSVKGRFAISRDNPPNTVYLQMNSLKPEDTAVYYCAKYPNNFWHNEAPDSWGRGTQVTVS
GST-6	QVQLVESGGGLVQPGESLRLSCAASGFTFSSHRMNWLRQAPGKGLEWVSTTSTGGGSAATYYVNSVKGRFTVSRDDAKNMLYLQMNSLKPEDTARYYCAIPESPGRPFGSTWSEYRYWGQGTQVTVS
GST-7	QVTLVESGGGLVQAGGSLGLSCTASGHTFNPYAMAWFRQAPGKEREFVAAIGWRVGTTYYTDSVKGRFTISADNAKNTAYLQMNSLKPEDTAVYYCAAGFGRSPSNYDYWGQGTQVTVS
GST-8	QVQLVESGGDLVQAGGSLRLSCAASGSISAAYDMGWFRQVPGKQRELVASLGSGGFSAYADSVKGRFTVSRDNAKNTVYLQMNSLKPEDTGVYYCAARTPFDIWTTGRWGEYDYWGQGTQVTVS
LaMIgG-5	MADVQLVESGGGSVQAGGSLRLSCAASGFIFSKDVMNWFRQAPGKERELVAFINPSGTTHYVDSVKGRFTISRDNVKSTVYLQMNDLKPDDTAVYYCQTRKFVGTTWRDYWGQGTQVTVS
LaMIgG-8	MAQVQLVESGGGLVQAGGSLKLSCATSGFTFSTSVMNWFRQAPGKERDMVAFINPSGSTNYVDSVKGRFTISRDSTKNTVYLQMNSLKPEDTAVYYCQTRLLVDSGWRDYWGQGTQVTVS

LaMIgG-9	MAQVQLVESGGGLVQAGGSLSLSCATSGRTGDLYAMAWFRQAPGAEREFV<br>ATVTAIGGATYYADSVKGRFTISASYARNMVYLQMNSLKPEDSAVYYCAASM<br>KRVWPLHRSDEAEYWGQGTQVTVS
LaMIgG-14	MAQVQLVESGGGLVQAGGSLRLSCAASGFTFSTSVMNWFRQAPGKEREM<br>VAFINPSGSTHYVDSVKGRFTISRDSAKNTVYLQLNSLKPEDTAVYYCQTRL<br>FVGAGWRDYWGQGTQVTVS
LaRIgG-1	MAQVQLAESGGGLVQPGGSLRLSCVVSGGTFGRYDMGWFRQAPGKEREF<br>VAAISREVTTNYGDSVRGRFTISRDNAENTVHLLMNSLKPEDTAVYYCAADD<br>AHRYSGTYYLSQDFRYTYWGQGTQVTVS
LaRIgG-2	MAQVQLVESGGGLVQAGGSLRLSCVASGGTFSDWPKGWFRQAPGKEREF<br>VAAINWNGSGTTYADSVKGRFTISRDNAKNAVYLEMNSLKPEDTAVYSCAST<br>FGGIRSVGTNDYWGRGTQVTVS
LaRIgG-7	MADVQLVESGGGLVQTGGSLRLSCAASGRTFTTYDLAWFRQAPGKERECV<br>AAVSRIGTTYYPDSVKGRFTISTDTAQNTVYLQMNSLKPEDTAVYYCASAKF<br>GSRWIRAKTSEGNYDDWGRGTQVTVS
LaGIgG-4	MADVQLVESGGGLVQAGGSLRLSCAASGITFSRSTMGWHRQAPGKRRELV<br>ASINAVGSTNYVPSVKGRFTISRDNAKNTVTLQMNSLEPEDTGVYYCVSDP<br>YDDPRRYWGPGTQVTVS
LaGIgG-12	MAQVQLVESGGGLVQAGGSLRLSCSASGRTLSTYAMGWFRQAPGKEREF<br>VAVISRSGGSTHYVESVKGRFTIVRDNAKNTVYLQMDSLKPEDTAVYYCAVP<br>KYTDYALSPEDFGSWGQGTQVTVS

Llama Magic 2.0

Detailed Methods

The Llama-Magic github repository is located at https://github.com/FenyoLab/Llama_Nanobodies. It includes all code, as well as detailed instructions for running the custom scripts for database preparation and for setting up and using the Llama-Magic web tool. Below is an outline describing the major steps in the pipeline.

Database Preparation

1) QC/Trim and Merge paired end sequencing reads: read pairs of length 300 bp are trimmed for quality and then merged. Trimmomatic (42) is used for trimming with parameters MAXINFO:275:0.8 (read 1) and MAXINFO:250:0.8 (read 2). Abyss (43) mergepairs is used for merging the reads with parameters -p 0.95 -m 25.

The following steps are performed using a series of custom perl scripts:

2) Convert merged FASTQ file to FASTA format.

3) Search for the PCR primers near the 5' and 3' ends of each merged sequence. This helps to determine the correct ORF for *in silico* translation, since the correct reading frame from the start position of the primer is known, and their orientation determines forward or reverse strandedness. This script adds the primer information to the header line of the FASTA file.

4) Perform an *in silico* translation to protein sequence. This script uses the output from step 3 to correctly perform the translation to protein sequence.

   (The previous version of the script performs all possible ORF translations and selects the longest as the correctly translated protein sequence. This is a simpler method; however, it does not always result in the correct ORF.)

5) Perform an *in silico* digestion of protein sequences with trypsin/chymotrypsin. One or both digestions can be performed. Allowed missed cleavages = 1 (trypsin) and 4 (chymotrypsin). This script produces a FASTA file where each unique (chymo-)tryptic peptide is listed on a separate line. It also produces an index file for input to the peptide mapping step (step 8). This index file lists the sequence ID and all unique peptides associated with that sequence for each sequence in the database.

<u>Llama-Magic: CDR finding, mapping of identified peptides and scoring of sequences</u>

6) Run X!Tandem(44): the input to X!Tandem is the converted raw instrument files (mgf format) and the pre-digested database from step 5. The output is an XML file with identified peptides, expectation scores and other descriptive information. Detailed parameter settings for the X!Tandem run in the form of an XML file can be found at the github repository.

7) Parse the XML output file of X!Tandem to a tab-delimited text file with the following columns. Only matches with log(e) < -1 are included.

Column name	XML tag/attribute
sequence	value of the **seq** attribute of the **domain** tag
log(e)	log10 of the value of the **expect** attribute of the **domain** tag
protein_uid	value of the **uid** attribute of the enclosing **protein** tag
domain_id	value of the **id** attribute of the **domain** tag
spectrum	text of the **note** tag with attribute **label="Description"**

8) **Perform peptide mapping and ranking of proteins**: map the peptides from step 7 (peptides from the *in silico* digested protein sequence database that were identified by X!Tandem) to the protein sequences from step 4, and calculate a score for each sequence based on the location and number of identified peptides it contains. The score is defined as:

$$8\,C_{CDR3} + 2\,C_{CDR2} + 2\,C_{CDR1} + 2C_{SEQ} + \frac{1}{15}L_{CDR3} + \frac{1}{10}L_{CDR2} + \frac{1}{9}L_{CDR1}$$

$$L = length\,of\,region$$

$$C\ (Coverage) = \frac{count\,of\,amino\,acids\,within\,an\,identified\,peptide}{L}$$

The sequences are sorted by score and grouped by CDR3 region (proteins with CDR3 regions of hamming distance = 1 are grouped together). The output produced by this step is an interactive HTML file that allows the user to view detailed information on the peptide

mapping for each protein sequence. Each peptide that maps to a sequence is shown as a clickable link that will allow the user to view the detailed MS2 coverage for the identification. In addition, a uniqueness score is calculated as:

$$Uniqueness = \frac{count\ of\ sequences\ in\ the\ group\ containing\ the\ peptide}{total\ number\ of\ sequences\ containing\ the\ peptide} x\ 100$$

If a user has performed both a trypsin and chymotrypsin digestion and has results from multiple MS/MS runs, it is possible to provide the results from both X!Tandem searches to this algorithm and it will combine the peptide identifications when mapping to the protein sequences and producing the sequence score.

<u>CDR finding:</u>

In order to score and rank the sequences, Llama-Magic implements a CDR finding algorithm as described in the table below. The listed positional indexing starts at the translated primer sequence starting position, which is where all the sequences start once they've been *in silico* translated (steps 3 & 4). For example, the indexing starts with the Valine at position 1 in this VHH sequence: 5'-**VLAAIGQG**VQAQVQVVESGGG…-3'. Llama-Magic clips the sequence at the 3' end following the sequence corresponding to the hinge region PCR primers: **PKTPKPQP** or **HHSEDP**. If the sequence of the short hinge is found, Llama-Magic will add 2 placeholder "amino acids"(**HHSEDP**XX) to keep the sequence lengths consistent for the CDR-finding algorithm.

The algorithm searches for certain conserved amino acid motifs at a range of positions to delineate the start and end of each CDR region as shown below. Alternate motifs are listed in subsequent lines and are searched if the previous motif is not found. When none

of the sequence motifs are found, default positions are used. Subsequent lines in the Default position column provide a last resort position for the case when neither the CDR start nor end position sequence motifs are found. The CDR3 region is first localized based on the ending position of the CDR2 region. If this fails, an earlier algorithm (from Llama-Magic 1.0) is used as an alternative (final 2 rows of table).

CDR position	Search positions	Sequence motif	Position of CDR st/end	Default position
$CDR1_{stpos}$	30 to 36	SC<br>C	$C_{pos}+5$	$CDR1_{endpos}-8$<br>38
$CDR1_{endpos}$	42 to 50	WXR<br>W	$W_{pos}-1$	$CDR1_{stpos}+8$<br>46
$CDR2_{stpos}$	42 to 50	WXR<br>W	$W_{pos}+14$	$CDR2_{endpos}-9$<br>61
$CDR2_{endpos}$	73 to 82	RF	$R_{pos}-8$	$CDR2_{stpos}+9$<br>70
$CDR3_{stpos}$	$CDR2_{endpos}$ to SEQ_{endpos}	DTAVYXC<br>DXAVYXC<br>DTXVYXC<br>DTAXYXC<br>DXXXYXC	$C_{pos}+3$	($CDR3_{stpos-alt}$)
$CDR3_{endpos}$	$CDR3_{stpos}$ to SEQ_{endpos}<br>$CDR2_{endpos}$ to SEQ_{endpos}	GTQVTV<br>XTQVTV<br>GXQVTV<br>GTXVTV<br>GTQXTV<br>GTQVXV<br>GTQVTX	$G_{pos}-4$	($CDR3_{endpos-alt}$)

$CDR3_{stpos-alt}$	102 to 112	YXC<br>C	C_{pos}+3	$CDR3_{endpos}$-11<br>SEQ_{endpos}-27
$CDR3_{endpos-alt}$	SEQ_{endpos}-20 to SEQ_{endpos}-10	WGQ<br>WG<br>W<br>XGQ	W_{pos}-1	$CDR3_{stpos}$+11<br>SEQ_{endpos}-16
